# *In vitro* differentiation of human embryonic stem cells into ovarian follicle-like cells

**DOI:** 10.1038/ncomms15680

**Published:** 2017-06-12

**Authors:** Dajung Jung, Jie Xiong, Min Ye, Xunsi Qin, Lin Li, Shunfeng Cheng, Mengyuan Luo, Jia Peng, Ji Dong, Fuchou Tang, Wei Shen, Martin M. Matzuk, Kehkooi Kee

**Affiliations:** 1 Center for Stem Cell Biology and Regenerative Medicine, Department of Basic Medical Sciences, School of Medicine, Tsinghua University, Beijing 100084, China; 2 Key Laboratory of Animal Reproduction and Germplasm Enhancement in Universities of Shandong, College of Animal Science and Technology, Qingdao Agricultural University, Qingdao 266109, China; 3 Tsinghua-Peking-NIBS Center for Life Sciences, School of Medicine, Tsinghua University, Beijing 100084, China; 4 Departments of Pathology & Immunology, Discovery, Baylor College of Medicine, Houston, Texas 77030, USA; 5 Department of Molecular & Human Genetics, Discovery, Baylor College of Medicine, Houston, Texas 77030, USA; 6 Biodynamic Optical Imaging Center & Department of Obstetrics and Gynecology, College of Life Sciences, Third Hospital, Peking University, Beijing 100871, China; 7 Department of Molecular & Cellular Biology, Discovery, Baylor College of Medicine, Houston, Texas 77030, USA; 8 Department of Pharmacology, Discovery, Baylor College of Medicine, Houston, Texas 77030, USA; 9 Center for Drug Discovery, Baylor College of Medicine, Houston, Texas 77030, USA; 10 Beijing Advanced Innovation Center for Structural Biology, School of Life Sciences, Tsinghua University, Beijing 100084, China

## Abstract

Understanding the unique mechanisms of human oogenesis necessitates the development of an *in vitro* system of stem cell differentiation into oocytes. Specialized cell types and organoids have been derived from human pluripotent stem cells *in vitro*, but generating a human ovarian follicle remains a challenge. Here we report that human embryonic stem cells can be induced to differentiate into ovarian follicle-like cells (FLCs) *in vitro*. First, we find that two RNA-binding proteins specifically expressed in germ cells, DAZL and BOULE, regulate the exit from pluripotency and entry into meiosis. By expressing DAZL and BOULE with recombinant human GDF9 and BMP15, these meiotic germ cells are further induced to form ovarian FLCs, including oocytes and granulosa cells. This robust *in vitro* differentiation system will allow the study of the unique molecular mechanisms underlying human pluripotent stem cell differentiation into late primordial germ cells, meiotic germ cells and ovarian follicles.

## Abstract (web summary)

*In vitro* production of human oocytes for the treatment of female infertility is a goal in reproductive medicine. Here, the authors establish *in vitro* conditions to generate human ovarian follicle-like cells from human embryonic stem cells.

To date, many studies have reported the successful derivation of male gametes from embryonic stem cells (ESCs)^1^,^2^,^3^,^4^,^5^,^6^,^7^, but only a few studies have reported the derivation of oocyte-like cells from pluripotent stem cells^8^,^9^,^10^. The first successful derivation reported was the spontaneous differentiation of mouse ESCs using medium containing fetal bovine serum (FBS)^9^, and the most recent method reported for obtaining oocyte-like cells in mice required the aggregation of primordial germ cells (PGCs) and somatic cells from E12.5 fetal gonads. This approach generated many oocyte-like cells, but the requirement of fetal ovarian tissues to obtain oocyte-like cells makes human studies technically challenging and may raise ethical issues. Therefore, a more complete *in vitro* differentiation approach that does not require the procurement of human tissue is desired.

Previous studies have shown that *Blimp1* and *Prdm14* are required for the differentiation of mouse epiblast stem cells into germ cells^11^,^12^. *In vitro* induction and overexpression of these intrinsic factors can direct mouse ESCs to differentiate into early germ cells, but meiosis is not initiated in these cells^13^. A recent study using human pluripotent stem cells reported similar conclusions regarding the role of PRDM1 in specifying human PGCs, but emphasized the different developmental mechanisms between the germ cells of humans and mice such as the differentially expressed SOX17 gene between the two species in PGC specification^14^.

Human embryonic stem cells (hESCs) are pluripotent cells that can differentiate into somatic and germ cell lineages. Exit from pluripotency and self-renewal has recently been discovered to be essential for ESC differentiation, and this process is directly regulated by a set of genes, including the gene encoding the RNA-binding protein PUM1 (refs 15, 16). The derivation of gametes from hESCs may also require similar regulators that govern the exit from pluripotency and the entry into a germ cell-specific state. For example, *Dazl* knockout mutants cannot undergo meiosis, and the expression levels of multiple pluripotency markers remain high^17^. *Dazl*, which encodes a germ cell-specific RNA-binding protein, has also been shown to directly regulate the expression of pluripotency markers during mouse ESC differentiation^18^,^19^. BOULE is another member of the DAZ protein family expressed in human germ cells^20^. A recent study indicated that BOULE is expressed in meiotic germ cells when DAZL is not expressed^21^, suggesting that BOULE may have an independent role in regulating meiosis that is not shared by DAZL. Previous study showed that DAZL is required for PGC formation during *in vitro* differentiation of hESCs, but its role in regulating pluripotency was not examined^4^.

In this study, we show that intrinsic factors DAZL and BOULE can modulate hESCs to exit pluripotency and enter into meiosis. Furthermore, extrinsic factors GDF9 and BMP15 can induce folliculogenesis in the differentiated hESCs. Transcriptome analysis, immunostaining of ovarian follicle markers and transplantation experiment all indicates that the follicle-like cells (FLCs) we derived resembling *in vivo* primordial follicle.

## Results

### DAZL regulates exit of pluripotency in human germ cells

**Figure 1:**
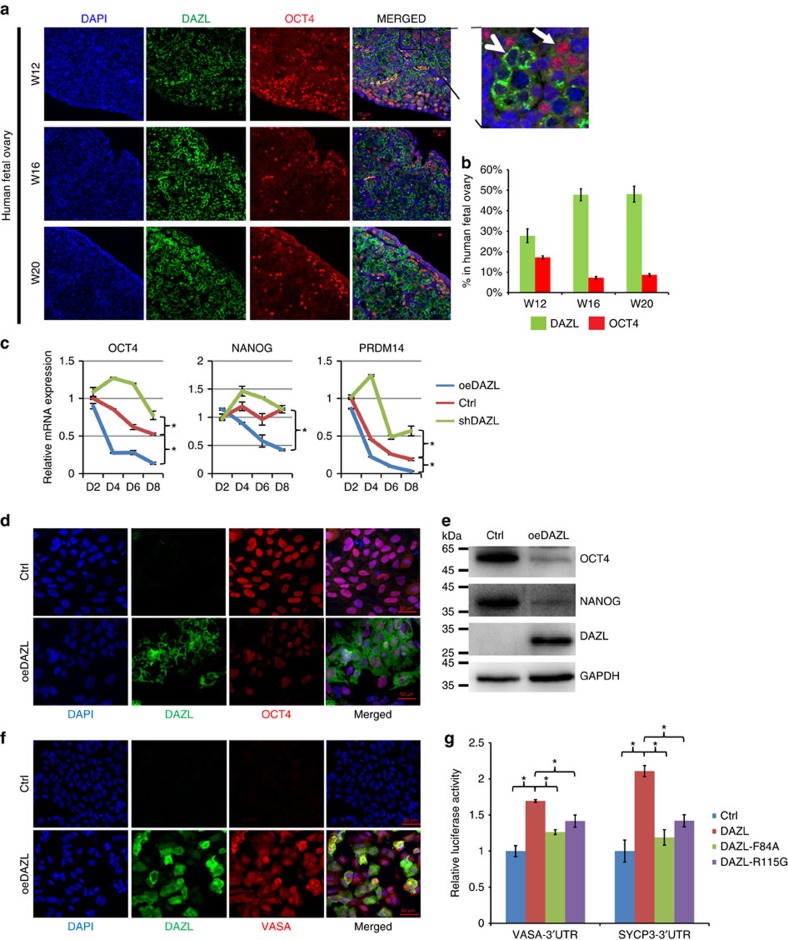
DAZL regulates the exit from pluripotency and entry into the late PGC stage. (**a**) Immunofluorescent staining of DAZL, OCT4 and DAPI (nuclei) in 12, 16 and 20 W human fetal ovaries. A magnified image is shown on the upper right corner. Bar, 10 μm. Arrow: OCT4-positive cells; arrow head: DAZL-strong positive cells. (**b**) Percentages of OCT4-positive cells, and DAZL-strong positive cells in the 12, 16 and 20 W human fetal ovaries. *n*=500–800 cells counted in three different fields, error bar is s.d. (**c**) Relative mRNA expression levels of OCT4, NANOG, PRDM14 measured by qPCR in the control (Ctrl), DAZL-overexpressing (oeDAZL) and DAZL-silenced (shDAZL), hESCs differentiated from day 2 to day 8. *n*=2 from each time point (replicates from ∼100,000 cells), three independent experiments conducted, error bar is s.d., asterisks indicate statistical significance (*P*<0.05, Student's *t*-test) between the two samples at the same time-points. (**d**) Immunofluorescent staining of DAZL and OCT4 in the Ctrl and oeDAZL hESCs at day 6. (**e**) Western analysis of OCT4, NANOG and DAZL in the Ctrl and oeDAZL hESCs at day 6. (**f**) Immunofluorescent staining of DAZL and VASA in in the Ctrl and oeDAZL hESCs at day 6. (**g**) 3′-UTR Luciferase reporter assays of VASA, and SYCP3 transfected with wild-type or the two DAZL mutants carrying point mutations F84A and R115G. *n*=4, (four independent biological replicates per cell populations, ∼25,000 cells in each sample), and the experiment had been conducted three times, error bar is s.d., asterisks indicate statistical significance (*P*<0.05, Student's *t*-test) between the two samples.

To determine whether DAZL regulates the exit from pluripotency in human germ cells, we first examined the expression of a pluripotent marker, OCT4, together with DAZL in human fetal ovaries collected from gestational week 12 to week 20 (Fig. 1a). Whereas the percentage of cells highly-expressing DAZL (arrow head in Fig. 1a) increased from 28 to 48% from W12 to W20, the percentages of OCT4-positive cells decreased from 17 to 9% (Fig. 1b). More importantly, cells expressing a high level of DAZL almost always lacked OCT4 expression, indicating a mutually exclusive expression pattern of the pluripotency marker and DAZL that is consistent with previous studies^22^. This finding suggests that DAZL may be responsible for the downregulation of pluripotency markers. To determine whether the increase in DAZL can down regulate the expression of pluripotency markers *in vitro*, we modulated the level of DAZL expression in hESCs during differentiation with bone morphogenetic proteins (BMPs) BMP4 and BMP8a (Fig. 1c). We found that 1 h of treatment with the BMPs induced the phosphorylation of pSmad1/5/8, indicating the differentiation of hESCs (Supplementary Fig. 1). To minimize the induction of other cell lineages during prolonged treatment with the BMPs, we treated hESCs for only 1 h before overexpressing DAZL. OCT4, NANOG and PRDM14 all exhibited significant decreases in their transcript levels from day 4 (D4) to day 8 (D8) when DAZL was overexpressed (using Student's *t-*test), but the expression levels of these genes remained at higher levels in the hESCs silenced with short hairpin RNA targeting DAZL (shDAZL). Although the expression of DAZL was low at this stage, the level of DAZL in the silencing sample was reduced to approximately half of the control (Supplementary Fig. 2). These findings indicate that many pluripotency markers are regulated by the level of DAZL expression in hESCs. The protein levels of the pluripotency markers, including OCT4 and NANOG (Fig. 1d,e), were also largely reduced in the differentiated hESCs expressing a high level of DAZL. By contrast, the protein level of the late germ cell marker VASA was greatly increased in the DAZL-overexpressing hESCs compared with the control hESCs (Fig. 1f). Taken together, these findings show that the increased expression of DAZL reduced the expression of pluripotency markers and increased the expression of a late PGC marker.

Recent studies have shown that DAZL can regulate multiple genes post-transcriptionally in mice^23^,^24^. Therefore, we investigated if DAZL regulates the expression of the late germ cell genes through the 3′-UTR region (Fig. 1g). We created two point mutations in the RNA-binding domain of DAZL. The R115G mutation was found to be associated with a premature ovarian failure patient^25^, and the F84A mutation was reported to affect the RNA binding of DAZL in a structural study^26^. The luciferase activities of VASA and the meiotic gene SYCP3 were significantly higher when DAZL was expressed compared with the controls (using Student's *t*-test). As expected, the mutants DAZL-F84A and DAZL-R115G induced significantly lower luciferase activities than wild-type DAZL. These results indicate that DAZL can regulate the expression of a late PGC gene and a meiotic gene through their 3′-UTRs. In addition, we confirmed that the point mutation R115G in DAZL has a detrimental effect on the posttranscriptional regulation of downstream genes.

### CDC25A is upregulated by BOULE in differentiated hESCs

**Figure 2:**
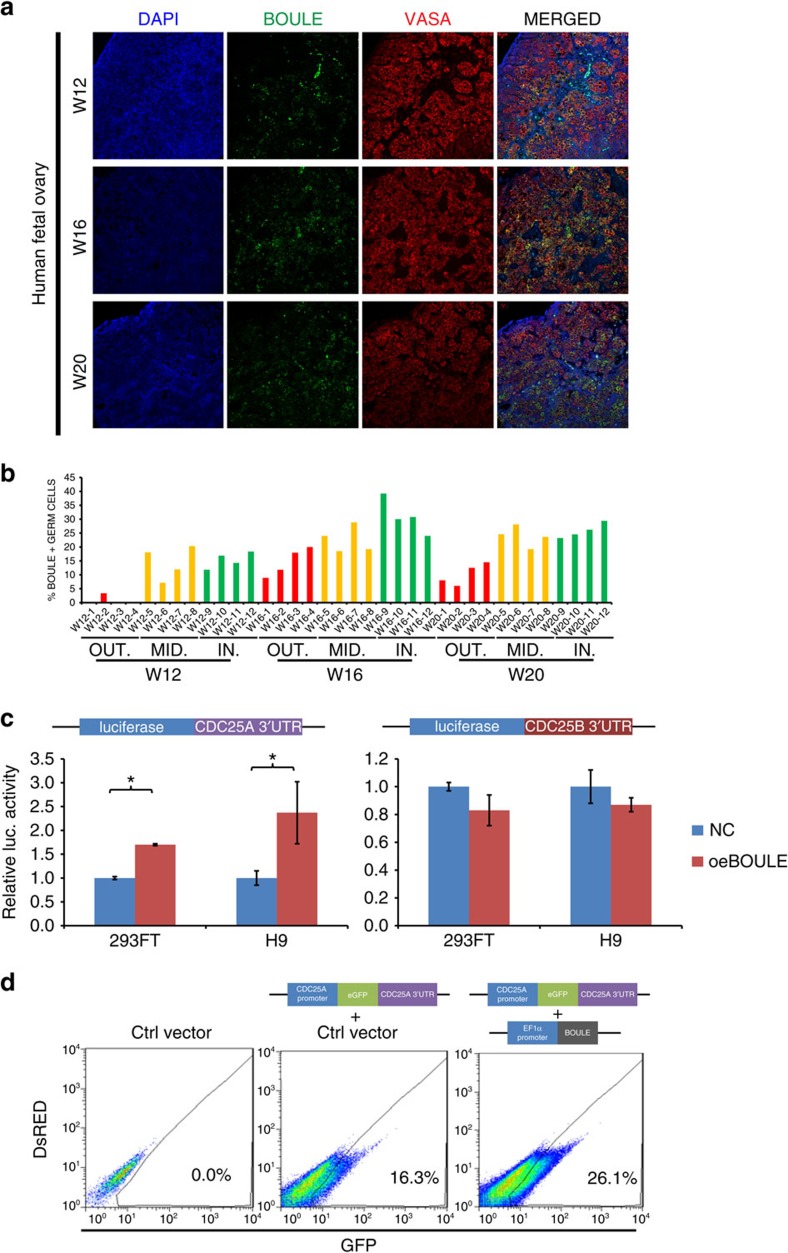
BOULE upregulated 3′-UTR of CDC25A in hESCs. (**a**) Immunofluorescent staining of BOULE, VASA and DAPI (nuclei) in 12, 16 and 20 W human fetal ovaries. Bar, 10 μm. (**b**) Distribution and location of BOULE-positive cells in cortex of human fetal ovaries. Percentages of BOULE-positive and VASA-positive cells in the 12, 16 and 20 W human fetal ovaries were counted as described in Supplementary Fig. 3. Areas of ovarian cortex were divided into outer (OUT), middle (MID) and inner (IN) sections. *n*=4 different fields were counted in each section as shown in Supplementary Fig. 3. Each area contained 50–117 VASA-positive germ cells. (**c**) 3′-UTR Luciferase reporter assays of CDC25A or CDC25B in 293FT or H9 hESCs. Cells were transfected with empty vectors (NC) or BOULE-overexpressing vectors (oeBOULE). *n*=3 (biological replicates from ∼25,000 cells), three independent experiments conducted, error bar is s.d., asterisks indicate statistical significance (*P*<0.05, Student's *t*-test) between the two samples. (**d**) FACS analysis of CDC25A promoter-eGFP-3′-UTR reporter assays in hESCs. CDC25A reporter was integrated into hESCs followed by transduction of empty vector (Ctrl vector) or BOULE-overexpressing vector(oeBOULE), representative FACS results were shown, 50,000 cells of control cells without reporter (left plot) and 100,000 cells of cells carrying CDC25A 3′-UTR reporter (middle and right plots) were analysed. Three independent experiments were conducted.

To examine the potential role of BOULE during meiotic progression, we first confirmed that the percentage of BOULE-positive germ cells reached the highest level in the inner cortex region (24–39%) of the W16 human ovary, compared with the W12 (12–18%) and W20 (23–29%) human ovaries (Fig. 2a,b, Supplementary Fig. 3). BOULE-positive germ cells appeared more frequently in the middle to inner cortex regions, consistent with the known distributions of early germ cells at the outer cortex and meiotic cells in the inner cortex (Fig. 2a,b). Previous studies have shown that an important cell cycle regulator in meiosis, Twine (CDC25 homologue in *Drosophila*), was regulated by Boule in *Drosophila*^27^, and CDC25A is an important regulator of meiosis I progression in mouse oocytes^28^. Therefore, we tested if BOULE regulates the luciferase activity through the 3′-UTR of CDC25A or CDC25B in human cells. Our results indicate that overexpression of BOULE specifically upregulated luciferase activity through the CDC25A 3′-UTR but not the CDC25B 3′-UTR, in both 293FT cells and H9 hESCs (Fig. 2c). In addition, when a gene reporter carrying the promoter and 3′-UTR of CDC25A was fused to the enhanced green fluorescence protein (eGFP) gene and transduced into hESCs, the percentage of eGFP-positive cells increased from 16.3 to 26.1% when BOULE was overexpressed in hESCs (Fig. 2d), showing CDC25A can be upregulated by BOULE in hESCs.

### DAZL and BOULE promote the entry into meiosis

**Figure 3:**
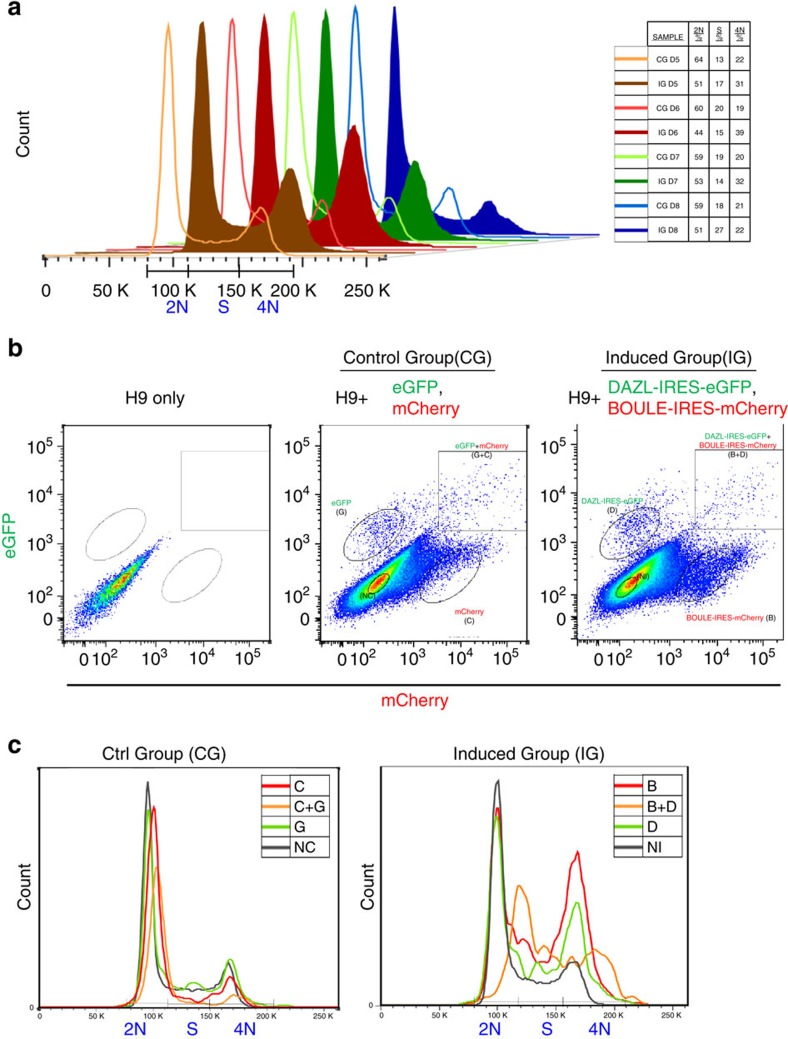
4n cells are enriched in cells with DAZL and BOULE overexpression. (**a**) DNA content analysis of hESCs transduced with control vectors eGFP and mCherry (control group, CG, line curves) or DAZL-IRES-eGFP and BOULE-IRES-mCherry vectors (induced group, IG, solid curves) from day 5 (D5) to day 8 (D8). In total, >500,000 cells were analysed for each sample. Right table shows corresponding percentage of 2n, S-phase and 4n cells in each sample. Three independent experiments were conducted. (**b**) FACS plots showing distinct populations expressing GFP, mCherry or both fluorescent proteins in either CG or IG groups. NC: cells without any fluorescent protein in CG, C: cells expressing mCherry, G: cells expressing eGFP, C+G: cells expressing mCherry and eGFP, NI: cells without any fluorescent protein in IG, B: cells expressing BOULE-IRES-mCherry, D: cells expressing DAZL-IRES-eGFP, B+D: cells expressing BOULE-IRES-mCherry and DAZL-IRES-eGFP. Cell populations were subdivided into different groups according to their expression of fluorescent proteins. (**c**) DNA-content profiles of subpopulations in CG and IG cells at D6.

By combining overexpression of DAZL and BOULE after BMPs treatment, we predicted that hESCs would proceed from a pluripotent hESCs into PGCs and enter into meiosis. Therefore, we next tested if meiosis can be induced *in vitro* in the differentiated hESCs. When cells enter meiosis, DNA content increases from 2n to 4n after DNA replication, and female oocytes are arrested in prophase I until puberty. If the treatment induced hESCs to enter prophase I of meiosis, there would be more 4n cells in the induced population versus control cells undergoing regular cell cycle. By FACS analysis of DNA content, we found that the 4n populations in the induced group (IG, overexpression of BOULE-IRES-mCherry, DAZL-IRES-eGFP) were higher than the control group (CG, overexpression of mCherry, eGFP) from D5 to D7 and peaked at D6 (Fig. 3a, 39% in IG and 19% in CG). Because the infected cells were a mixed population of different combinations, the differentiated hESCs were further divided by FACS (Fig. 3b) into no overexpression (NI), BOULE-IRES-mCherry overexpression (B), DAZL-IRES-eGFP overexpression (D) and BOULE-IRES-mCherry+DAZL-IRES-eGFP overexpression (B+D). The DNA content profile of B, D and B+D showed higher numbers of S-phase and 4n cells than the NI group. This was not due to overexpression of mCherry or eGFP fluorescent proteins, as the corresponding groups in CG all showed similar profiles of low 4n cell frequency (Fig. 3c). Among these three groups, B+D group showed the lowest numbers of 2n cells and the highest number of S-phase plus 4n cells from D4 to D8 (Supplementary Fig. 4), suggesting that the cells with BOULE and DAZL overexpression were entering meiosis and replicating their chromosomes.

**Figure 4:**
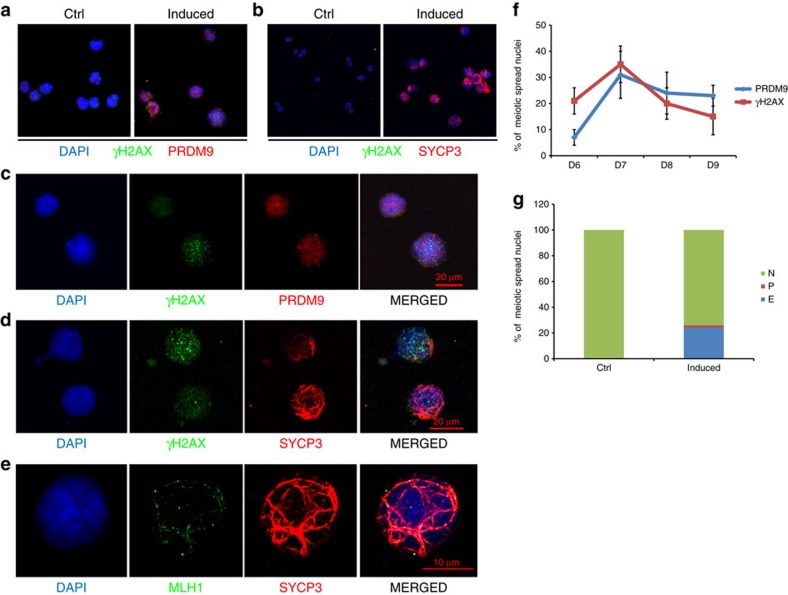
Meiotic progressions in the differentiated hESCs. (**a**) Representative images of meiotic spreads and immunofluorescent staining of PRDM9 and γH2AX in control and induced hESCs. (**b**) Representative images of meiotic spreads and immunofluorescent staining of SYCP3 and γH2AX in control and induced hESCs. (**c**) Magnified image of the meiotic spreads and the immunofluorescent staining of PRDM9 and γH2AX in induced hESCs. Bar, 20 μm. (**d**) Magnified image of the meiotic spreads and immunofluorescent staining of SYCP3 and γH2AX in induced hESCs. Bar, 20 μm. (**e**) Magnified image of the meiotic spreads and immunofluorescent staining of SYCP3 and MLH1 in induced hESCs. Bar, 10 μm. (**f**) Percentages of PRDM9- and γH2AX-positive nuclei during *in vitro*-induced meiosis from day 6 to day 9, *n*>200 meiotic nuclei at each time point, error bar indicates s.d. (**g**) Percentages of nuclei with no SYCP3 (N), punctate SYCP3 (P) or elongated SYCP3 (E) during *in vitro*-induced meiosis at day 7, *n*>200 meiotic nuclei. At least three independent experiments were conducted for each set of meiotic spreads and immunostainings described above.

To confirm that the induced cells had entered meiosis, we examined the protein expression and subcellular localisation of important meiotic markers. We prepared meiotic spreads of the differentiated hESCs and stained the spreads for PRDM9 and γH2AX. PRDM9 is a meiotic recombination-site determinant and is known to appear in meiotic nuclei at approximately the preleptotene stage^29^. γH2AX is known to appear when meiotic DSBs are initiated^30^. Nuclei positive for PRDM9 and γH2AX started to appear at D6 in the induced cells but not in control cells without induction (Fig. 4a,c). The γH2AX expression level varied among the PRDM9-positive nuclei, but the percentage of nuclei positive for either PRDM9 or γH2AX reached ∼30% at D7 and decreased at D8 and D9 (Fig. 4f). The similar changes of the percentages of nuclei positive for PRDM9 or γH2AX during the D6–D9 period suggests that there is a transient occurrence of meiotic DSBs *in vitro* in the induced hESCs, consistent with the transient appearance of high 4n cells as determined by FACS analysis. Synaptonemal complex (SC) formation is another hallmark of meiotic progression in germ cells. A component of the SC, SYCP3, appeared in an elongated pattern in many of the nuclei of the induced hESCs but not the control cells (Fig. 4b,d, Supplementary Fig. 5). As expected, most nuclei containing elongated SYCP3 expressed less γH2AX, suggesting that meiosis had proceeded to a later stage (Fig. 4d). At D7, we detected ∼20% elongated SYCP3-positive nuclei in the induced hESC population (Fig. 4g). MLH1 is a meiotic-specific protein that participates in DNA repair at the recombination nodules and appears after meiotic initiation^31^. Its punctate staining pattern has been shown to be closely associated with the SC in pachytene nuclei. At D8, we observed some nuclei showing punctate MLH1 expression that colocalized with or was close to the SYCP3 thread-like structure (Fig. 4e). In summary, we observed the appearance of multiple meiotic markers in the induced hESCs starting from D6 to D9, and together with the DNA content analysis, our results indicate that hESCs can be induced *in vitro* to enter meiosis with the addition of BMPs, followed by the overexpression of key intrinsic factors, specifically DAZL and BOULE (Supplementary Fig. 6).

### GDF9 and BMP15 induce ovarian follicle formation in hESCs

**Figure 5:**
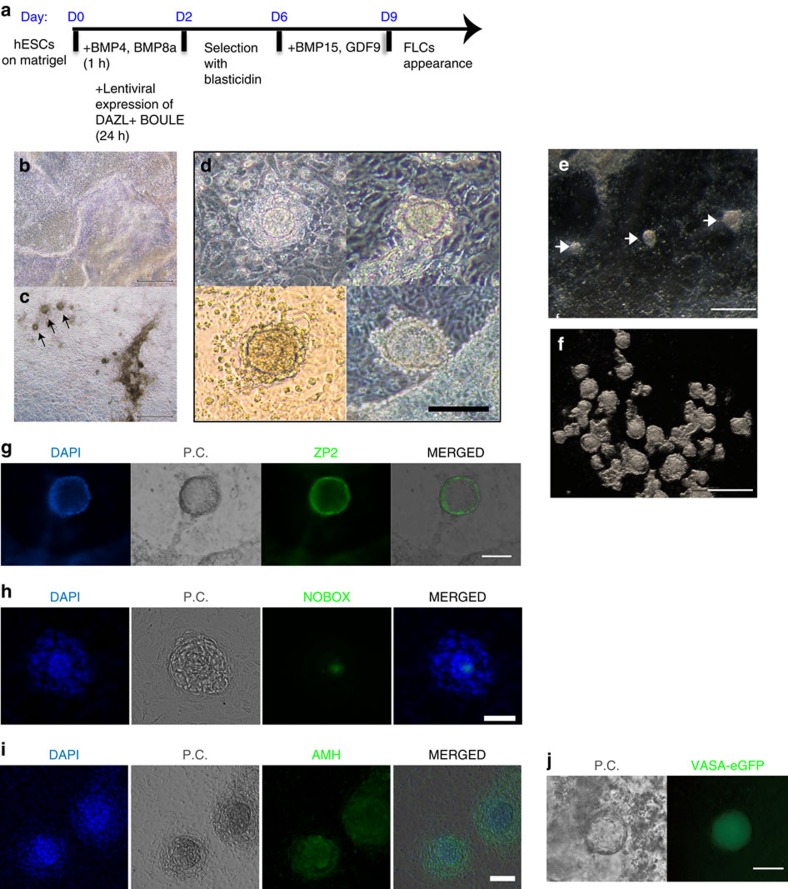
Appearance of ovarian FLCs induced in differentiated hESCs. (**a**) Schematic timelines and simplified protocols for the differentiation from the undifferentiated hESC stage to the appearance of FLCs. (**b**) Phase-contrast image of a differentiated hESC culture without inducers at day 11. (**c**) Phase-contrast image of a differentiated hESC culture with the inducers described in **a** at day 11. (**d**) Magnified phase-contrast images of four different FLCs appearing at day 11 in the induced culture. (**e**) Stereo-microscope image of a differentiated hESC culture with inducers at day 11. (**f**) Stereo-microscope image of a group of FLCs collected from a differentiated hESC culture with inducers at day 11. Immunofluorescent staining of ovarian follicle markers ZP2 (**g**), NOBOX (**h**) and AMH (**i**) on the culture plate with FLCs. (**j**) VASA-eGFP reporter cells specifically expressed eGFP in cells that became FLCs. P.C.: phase contrast. Bar, 500 μm in **b**,**c**,**e**,**f**; 100 μm in **d**; 50 μm in **g**,**h**,**i**,**j**.

The initiation of meiosis is followed by folliculogenesis in the mammalian ovary, but the development of the ovarian follicle requires many autocrine and paracrine factors, such as GDF9 and BMP15 (ref. 32). We tested if incubation with both human recombinant GDF9 and BMP15 can induce folliculogenesis *in vitro*. After induction with the BMPs and overexpression of DAZL and BOULE, we added recombinant GDF9 and BMP15 to the differentiation medium at D7 (Fig. 5a). FLCs started to appear around D9 but not in the control hESCs differentiated without overexpression of DAZL and BOULE or in the absence of GDF9 and BMP15 (Fig. 5b–d). The morphology of the FLCs under a phase-contrast microscope appeared as a multilayered-cell aggregate surrounding an oocyte-like cell in the middle (Fig. 5d, Supplementary Fig. 7), with sizes ranging from 50 to 100 μm. Under a stereo-microscope, these FLCs appeared much less transparent, so we collected them as suspended cell clumps (Fig. 5e,f). The appearance and number of FLCs were periodic and reproducible, that is, the number of FLCs peaked ∼D11–D13 and diminished after D15 (Supplementary Fig. 8). On average, there were ∼10 FLCs per four wells at D11 to D13. ZP2 protein, a component of the zona pellucida, was clearly detected and was localized specifically at the periphery of the oocyte-like cell in the middle of FLCs (Fig. 5g). Furthermore, the oocyte-specific transcription factor NOBOX, which is known to be expressed at the nucleus of the oocyte, was detected at the centre of the FLCs^33^ (Fig. 5h). AMH, a granulosa/cumulus cell marker, was expressed around the oocyte-like cells in multiple layers (Fig. 5i). Hence, at approximately D11, not only did some of the differentiated hESCs show characteristics of oocytes, but their surrounding cells resembled granulosa cells. When hESC carrying the VASA-eGFP reporter^4^ were differentiated with the same inducers and procedures, eGFP was specifically expressed in the middle of the FLCs, indicating the oocyte property of the central cell (Fig. 5j).

### FLCs transcriptome indicates its primordial follicle identity

**Figure 6:**
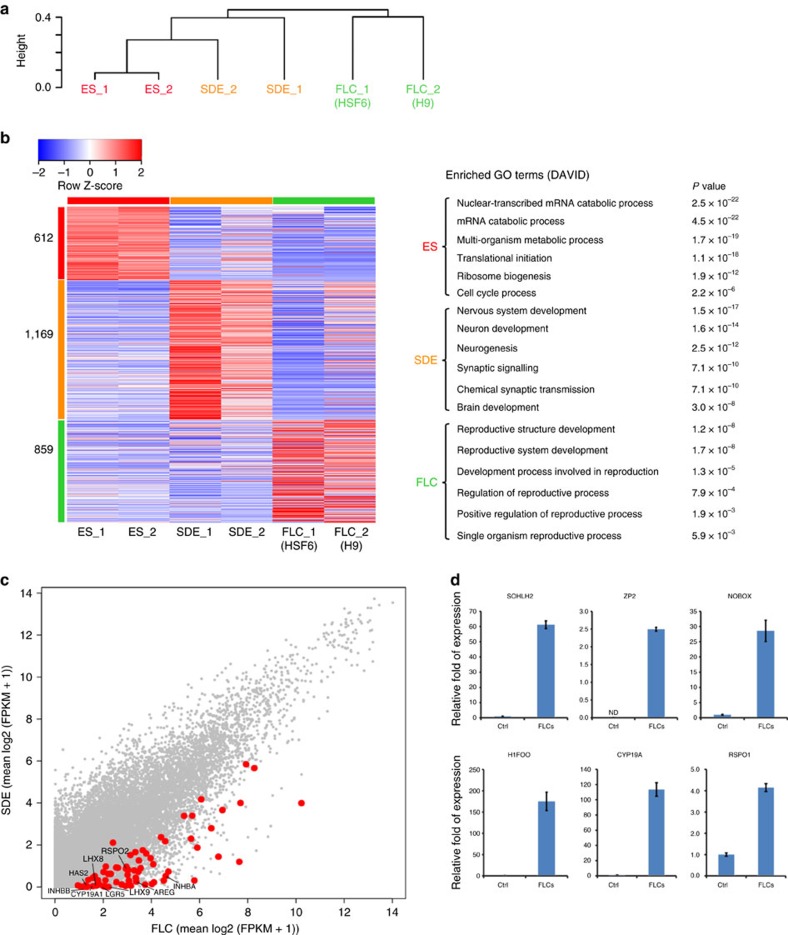
Transcriptional profiles of FLCs by whole-genome RNA sequencing and qPCR. (**a**) Unsupervised hierarchical clustering of gene expression in hESC (ES), spontaneously differentiated hESCs (SDE) and FLCs (one sample from H9 and one from HSF6). Independent biological duplicates of each group were subjected to whole-genome RNA sequencing. (**b**) Heat map of gene expression of ES, SDE and FLC shows different expression of genes in these three groups. Left, heat map; right, gene ontology (GO) of 612, 1169 and 859 differentially expressed genes. (**c**) Scatter plot analysis of transcripts between SDE and FLC. Red dots show genes enriched in reproductive tissues (oocytes or granulosa cells) that are highly expressed in FLC. (**d**) mRNA expression of SOHLH2, ZP2, NOBOX, H1FOO, CYP19A and RSPO1 was evaluated by an independent experiment collecting 30 FLCs compared with the same number of cells from control cultures. *n*=3 (technical replicates of 30 independent FLCs collected and pooled together), error bar indicates SD, >3 independent experiments.

To determine whether the FLCs expressed any gene marker of ovarian follicles, we performed whole-genome transcriptome analysis of the undifferentiated hESCs (ES), spontaneously differentiated hESCs (SDE), and FLCs differentiated from two independent hESC lines (H9 and HSF6). Unsupervised hierarchical clustering of genes showed that each group clustered to its duplicate and ES clustered closer to SDE than to FLCs (Fig. 6a). A heat map of these three groups showed many differentially expressed genes, which were grouped according to gene ontology into three groups of 612, 1,169 and 859 (Fig. 6b, Supplementary Data 1). Among the 859 FLCs genes, there are many genes, which are involved in reproductive processes, versus the 1,169 SDE genes, which are more likely to be involved in neuron development. A scatter plot of transcripts between the SDE and FLC groups showed that many genes known to be involved in reproductive processes were enriched in FLCs (Fig. 6c). Principal component analysis of our transcriptome datasets together with the previously published transcriptome studies of human primordial oocyte, MII oocyte and granulosa cells^34^,^35^ showed that our FLCs clustered closer to primordial oocyte than MII oocyte or granulosa cells (Supplementary Fig. 9). Furthermore, independent qPCR analysis of control versus FLCs confirmed that oocyte-specific markers SOHLH2, ZP2, NOBOX and H1FOO and the genes CYP19A and RSPO1, expressed in granulosa cells, were all highly expressed in the FLCs compared with the controls (Fig. 6d). The specific appearance of FLCs in the induced hESCs with intrinsic factors (DAZL and BOULE) and extrinsic factors (GDF9 and BMP15) suggested that the hESCs need to exit from the pluripotent state, differentiate to late germ cells and enter meiosis before folliculogenesis can be induced by the extrinsic factors.

### Transplanted FLCs resemble primordial follicle

**Figure 7:**
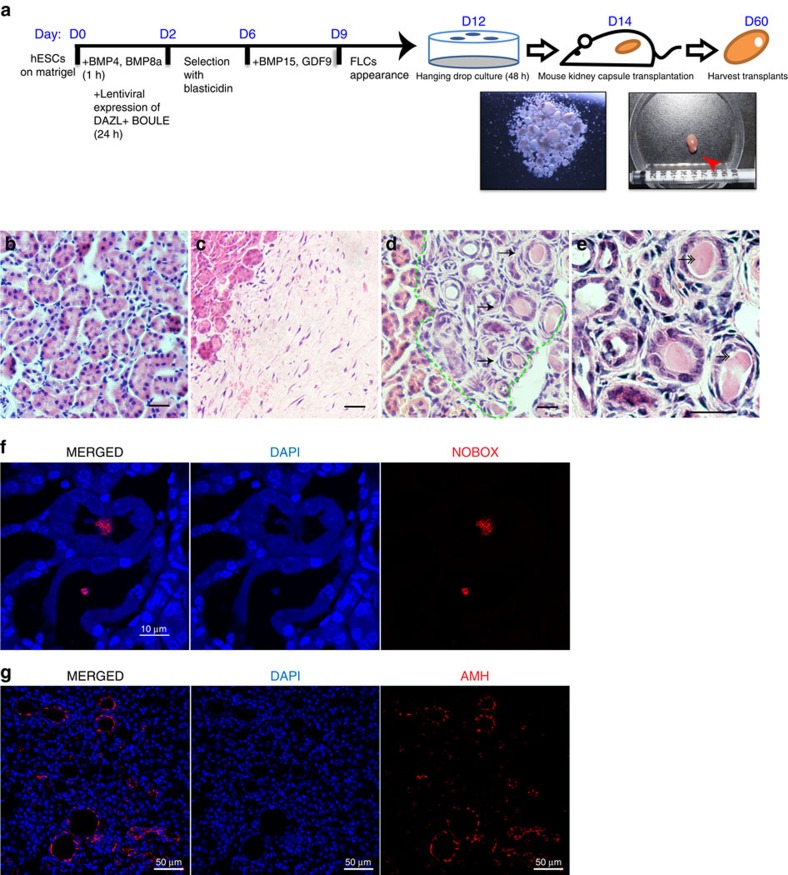
Existence of ovarian FLCs after transplantation into mouse kidney capsules. (**a**) Schematic timelines and simplified protocols for the differentiation of the undifferentiated hESCs to the harvesting of the transplant from mouse kidney capsules. Red arrow: harvested transplant. Representative images of H&E staining of a kidney area next to the transplant (**b**), a control transplant containing differentiated hESCs without inducers (**c**), a transplant containing suspended FLCs as described in (**d**), and a magnified picture of FLCs in the transplant (**e**). Arrows: FLCs with primary follicle-like structures; double-headed-arrows: GV-like staining in the FLCs. (**f**) Immunofluorescent staining of NOBOX in the transplant showing colocalisation of NOBOX and nuclei in the middle of follicles. (**g**) Immunofluorescent staining of AMH in the transplant showing the AMH-positive cells localized to the surrounding cells of follicles. Bar, 50 μm in **b**–**e**,**g**; 10 μm in **f**. Three independent transplantation experiments were conducted.

To further confirm that the FLCs obtained in our culture were ovarian follicles, we transplanted these FLCs into mouse kidney capsules. After collecting FLCs from the culture plate and culturing via hanging drop technique for 48 h, the aggregated cell clumps were transplanted into a kidney capsule for 46 days (Fig. 7a). Many ovarian follicle-like structures appeared in the transplants, but not in the control transplants or the surrounding kidney tissues (Fig. 7b–d). The cuboidal morphology of the surrounding cells and the germinal vesicle-like staining in the middle of the oocyte-like cells suggested that these FLCs were primary follicles (Fig. 7e). More importantly, immunostaining of the transplant confirmed that these FLCs expressed NOBOX and AMH at the expected cellular locations, specifically the nucleus of the oocyte and the surrounding granulosa cells, respectively (Fig. 7f,g, Supplementary Fig. 10). Therefore, the FLCs derived *in vitro* maintained their ovarian follicle properties even in the transplanted environment, confirming the identities of these cells. In addition, we tested if these FLCs secreted estradiol as a signature of folliculogenesis. After incubation with *in vitro* maturation medium as reported previously^36^, we found that estradiol could be detected from 1 to 6 days in the drop culture supernatant containing FLCs (Supplementary Fig. 11), but the level of estradiol is below accurate detection in the control supernatant.

## Discussion

The recent success of differentiating mouse ESCs into oocyte-like cells has encouraged many scientists to attempt a similar approach in humans. However, the underlying developmental mechanisms may be substantially different between the mouse and human, which might hinder the successful use of derivations of human oocyte-like cells. The most recent success of differentiating mouse ESCs to oocyte still requires aggregation of somatic cells from fetal ovaries^10^. This would not be feasible if ones want to obtain oocyte from hESCs using the same approach. The AMH-positive cells in our differentiated hESCs may be induced by the neighbouring germ cells and the extrinsic factors, GDF9 and BMP15, we added to the culture.

Two groups have reported *in vitro* differentiation of hESCs to germ cells, and SOX17 was found to act as a transcriptional factor that regulates human PGC specification but not in mouse PGC specification^14^,^37^. The differences of transcriptional profiles and regulations between mouse and human PGCs highlight the importance of using human-cell based approach to study human germ cell development.

Our approach of inducing hESCs to the late PGC stage and inducing meiosis and folliculogenesis advances the progress in obtaining human oocytes derived from pluripotent stem cells *in vitro*. Previous studies have revealed the potential of DAZL as a key regulator of germ cell development in mice, especially during the initiation of meiosis^17^. Here, we showed that a combination of DAZL and BOULE can be utilized to induce hESCs to exit the pluripotent state and enter meiosis *in vitro*, supporting a model that there are some RNA-binding proteins that act as key regulators of germ cell progression in the late germ cell and meiosis (Supplementary Fig. 6), and confirming the recent prediction that DAZL has an important role in human female fertility^38^. It is interesting that PUM1, another RNA-binding protein, has similar role in regulating the exit of pluripotency by destabilising the mRNA of the target genes known to be essential for pluripotency circuitry^15^. Perhaps there is a common mode of regulating exit of pluripotency using RNA-binding proteins at the posttranscriptional level in various cell types.

We further tested if extrinsic factors known to be important in mice could be utilized in inducing human folliculogenesis *in vitro*. Combining recombinant GDF9 and BMP15 prepared as heterodimers as previously described^39^, the meiotic germ cells promoted by DAZL and BOULE were induced to initiate folliculogenesis *in vitro*. We noted that the FLCs contained an oocyte-like cell in the middle of the cell aggregate as well as granulosa-like cells at the outer layer, suggesting that the mixed population of cells in the differentiated culture had the ability to self-organize into an ordered biological entity. This property has been reported in many other organoids differentiated from ESCs, including optic-cup and cerebral organoids^40^,^41^, but this is the first report of follicle-like structure resembling primary follicle derived from hESCs. Taken together, the *in vitro* differentiation system reported here showed that the hESCs exited pluripotency, progressed into meiosis, and initiated folliculogenesis. This system can be used to investigate genes or mechanisms that are unique in human germ cell development such as the functional roles of lncRNAs, which had low conservations among species.

## Methods

### hESC culture and induction of folliculogenesis

The hESC line H9 (female XX line) was purchased from WiCell, Inc., and HSF6 (female XX line) was a gift from Renee Reijo Pera's laboratory. Both lines are propagated as described previously^42^. In brief, undifferentiated hESCs were expanded on irradiated MEFs at 37 °C with 5% CO_2_ in knockout DMEM (KODMEM; Invitrogen) with knockout serum replacer plus bFGF medium (20% knockout serum replacer, 1 mM L-glutamine, 0.1 mM nonessential amino acids, and 8 ng ml^−1^ recombinant human basic FGF (R&D systems)). To differentiate hESCs into ovarian follicle cells, ∼5 × 10^4^ undifferentiated hESCs (∼50% confluency in a six-well plate) were seeded onto one well of a six-well plate coated with Matrigel. Adherent differentiation began by treating the hESCs with differentiation medium (KODMEM supplemented with 10% FBS, 1 mM L-glutamine, 0.1 mM nonessential amino acids, 50 ng ml^−1^ BMP4 and BMP8a (R&D systems)) for 1 h at 37 °C with 5% CO_2._ This procedure was followed by transduction with lentiviral supernatant expressing DAZL and BOULE for 1 day, recovery for 1 day, selection for 3 days with blasticidin at 2 μg ml^−1^, and recovery for another day. At day 7, the differentiated hESCs were incubated with differentiation medium containing 0.35 ng ml^−1^ GDF9:BMP15 heterodimer (prepared as described previously in Matzuk's lab^39^) and 10 ng ml^−1^ EGF (R&D) for 1 day, followed by new differentiation medium containing 50 ng ml^−1^ GDF9 and 25 ng ml^−1^ BMP15 (R&D systems). Thereafter, the differentiation medium containing 50 ng ml^−1^ GDF9 and 25 ng ml^−1^ BMP15 was replaced every 3 days until FLCs appeared or the cells were harvested.

### Human fetal ovary tissues

Human fetal ovaries were procured from Tsinghua University Second Hospital with the approval of the institutional review boards (IRB) of the hospital and the IRB of the School of Medicine, Tsinghua University. All fetal ovaries were collected from elected abortions with consent forms from the patients, and the patient information was kept confidential from the researchers. All tissues were immediately fixed upon collection and were used for histological analysis in this project without any further expansion or derivations of living cells.

### Histological analysis and immunofluorescent staining

The human fetal ovaries were dissected, fixed in 4% paraformaldehyde overnight and embedded in paraffin using standard protocols. Transplants collected from the kidney capsule were also fixed overnight and embedded as described above. The paraffin embedded tissues were slices into 5 μm sections and dried overnight. Sections were dewaxed, rehydrated and stained in hematoxylin and eosin. To perform immunofluorescent staining, sections were retrieved in 10 mM Sodium Citrate buffer, pH 6.0, for 5 min at 80 °C in a microwave on stew mode with another 20 min in the microwave prior to cooling in the buffer at room temperature. Sections were washed in Tris-buffered saline plus tween-20 (TBST; 0.5 M Tris, 1.5 M NaCl, tween-20 0.1%, pH 7.6) three times, 5 min each wash at 100 r.p.m. on a horizontal shaker. Then, sections were blocked in TBST with 10% donkey serum (BIODEE, China) for 1 h followed by incubation with the primary antibody; the dilutions are listed in Supplementary Table 1. Sections were washed in TBST three times for 5 min followed by incubation in the secondary antibody at the indicated dilution listed in Supplementary Table 1 for 1 h at room temperature. Sections were stained with DAPI after three TBST washes for 5 min. A drop of ProLong Gold Antifade (Life Technologies) was used to mount the section with a coverslip. Images were collected using a confocal microscope (Nikon).

hESCs (H9) were grown to adhere on cover slips coated with 1% Matrigel. After the hESCs were differentiated as described above, the adherent differentiated cells were rinsed twice in phosphate-buffered saline (PBS), pH 7.4, fixed with 4% paraformaldehyde for 10 min, and permeabilized with 0.25% Triton X-100 in PBS for 15 min at room temperature. Permeabilized cells were blocked in 10% donkey serum in PBS at 4 °C for 1 h with gentle agitation. The blocked cells were then incubated overnight at 4 °C with the primary antibody at the dilution listed in Supplementary Table 1. The cover slips were washed with PBS three times and then incubated with the secondary antibody listed in Supplementary Table 1 for 1 h at room temperature. Then, the cover slips were washed as described above, stained with 1 μg ml^−1^ DAPI for 30 min at room temperature, and then washed again as described above. The slides were mounted with ProLong Gold Antifade (Life Technologies). The slides were observed under a confocal microscope.

### Overexpression and silencing of genes in hESCs

Overexpression and silencing vectors of DAZL and BOULE were constructed and tested previously^42^. shDAZL was shDAZL4 carrying the targeting sequence: GCATTTCCTGCTTATCCAAAT. The overexpressing DAZL mutant, DAZL-F84A and DAZL-R115G were constructed by overlapping PCR to generate the same point mutations described in previous studies^25^,^26^ and were then inserted into the lentiviral vector p2k7 as the wild-type DAZL we used in our previous study.

### Dual-luciferase reporter assay

The 3′-UTRs of human VASA and SYCP3, according to the sequences downloaded from the NCBI website (primer sequences are listed in Supplementary Table 2), were cloned into the psiCHECK2 vector (Promega, C8021). One day before transfection, 293FT cells were transferred to a well in a 24-well plate at a density of 1 × 10^4^ cells per well. The next day, the 293FT cells were transfected in replicates of four with luciferase vectors carrying individual 3′-UTRs and an overexpression p2k7 vector carrying either DAZL, DAZL-R115G or DAZL-F84A or an empty vector using Lipofectamine 2000 (Life technologies, 11668019) in Opti-MEM medium (20 ng dual-luciferase vector and 160 ng overexpression vector). After 6 h of transfection, the transfection medium was replaced with 293FT medium and incubated at 37 °C for 48 h. To measure the luciferase activity, the transfected cells were lysed and assayed using the Dual-luciferase Reporter Assay System according to the manufacturer's protocol (Promega, E1960). The relative luciferase activity was calculated by first normalising the values to the Renilla/firefly luciferase in the cells transfected with psiCheck2 empty vector followed by normalisation to the cells transfected with the empty overexpression vector.

### Western blot analysis

The procedure for the western blot analysis was previously described^42^, and the specific antibodies are listed in Supplementary Table 1. In brief, the differentiated cells were washed with 3 ml cold PBS w/o Ca^++^ and Mg^++^, then scraped from the plate in 1 ml PBS plus 2 × protease inhibitors (Complete Mini, Roche) and transferred immediately to a 1.5 ml tube on ice. The cell suspension was then spun at 5,000 r.p.m. in a microcentrifuge for 3 min and the supernatant was discarded. The cell pellet was resuspended with 200 μl RIPA buffer (50 mM Tris, 150 mM NaCl, 0.5% Sodium deoxycholate, 1% NP-40, 0.1% SDS, pH 8) plus 2 × protease inhibitors (Complete Mini, Roche). The cell pellet suspension was pipetted rigorously at least 10 times, then vortexed for 30 s. The suspension was again spun down for 3 min at the same speed. The supernatant was measured for protein concentration and denatured in Laemmli buffer at 95 °C for 5 min, then loaded onto either a 10% or 12% SDS-PAGE gel. The SDS-PAGE gels were run at 150 volts for 1 h and transferred to a PVDF membrane for 1 h at 100 volts in CAPS buffer (10 mM CAPS, 10% methanol, pH 11). Transferred blots were blocked in 5% non-fat milk for 1 h at room temperature. The blot was subjected to 1 h of primary antibody incubation, followed by two quick rinses and three washes for 5 min in TBST (TBS, pH 7.4 with 0.1% Tween-20). Secondary antibody incubation had the same duration and washes. ECL+ (Amersham) was used to detect the HRP signal and the western blot image was collected using Chemidoc XRS (Bio-Rad). Uncropped western blots are shown in Supplementary Fig. 12.

### Quantitative PCR

Total RNA was extracted using the RNeasy Mini Kit (Qiagen) and the cDNA was synthesized using the iScript cDNA Synthesis Kit (Bio-Rad) according to manufacturer's protocols. Quantitative PCR was performed using the iTaq Universal SYBR Green Supermix (Bio-Rad) on a CFX96 Real-time PCR machine (Bio-Rad). The gene expression level was first normalized to the housekeeping gene glyceraldehyde 3-phosphate dehydrogenase and then normalized to the expression of a control sample in each set of experiments using Bio-Rad CFX Manager program and relative expression formulation dC(t)^43^. Statistical analysis was performed using GraphPad Prism 5. Real-time PCR primers for all gene sequences are listed in Supplementary Table 2.

### Whole-genome transcriptional profiling of FLCs by RNA-Seq

Each FLC was picked out from the plate and lysed in 1 ml TRIzol reagent (Invitrogen, 15596026). 100 μl BCP (MRC, BP151) was added into the lysate, vortexed for 15 s, waited for 3 min at RT, then centrifuged for 15 min at 16,000 × *g* at 4 °C. The supernatant was transferred into a new tube followed by adding one volume of isopropanol and 20 μg glycogen, then precipitated at −20 °C overnight. The total RNA was centrifuged at 16,000 × *g* for 15 min at 4 °C, the precipitate was washed twice by 75% ethanol, then dissolved in RNase-free water. A total amount of 125 ng RNA of each sample was used for RNA-Seq. DNA library was prepared according to the protocol of NEBNext Ultra DNA Library Prep Kit for Illumina (NEB, E7370). The final quality-ensured libraries were pooled and sequenced on the Illumina HiSeq2000/2500 sequencer for 100 or 150 bp paired-end sequencing^44^.

### DNA content analysis

hESCs were first seeded at 50% confluency onto six-well-plate coated with Matrigel. To induce differentiation, adherent cells were treated with 50 ng ml^−1^ BMP4 and BMP8a for 1 h in differentiation medium and followed by transduction of lentivirus expressing DAZL-IRES-eGFP and BOULE-P2A-mCherry for 1 day, recovery for 1 day, selection with blasticidin at 2 μg ml^−1^ for 3 days, and recovery for another day. Thereafter, cells were treated with differentiation medium until day 8. Differentiated cells were collected into a 15 ml tube, washed once with 1 ml chilled PBS, and suspended in 5 μg ml^−1^ Hoechst 33342 (Beyotime) and 20 μM verapamil hydrochloride (Sigma). Cell suspension was incubated at 37 °C for 40 min. During the incubation, tubes were mixed several times. The cells were resuspended in 20 μM verapamil hydrochloride (Sigma) and analysed by LSRFortessa SORP (BD). A total of >500,000 cells from each sample were subjected to DNA content analysis using programs in FlowJo 7.6 software package.

### Meiotic spreads and meiotic markers immunofluorescent staining

Meiotic spreads were conducted as described previously with some modifications^42^. Induced hESCs were collected as a single cell suspension from one well of a six-well plate, centrifuged, resuspended in hypotonic extraction buffer and dropped onto slide glasses dipped in 1% paraformaldehyde. The slide glasses were kept overnight in a humidified box at room temperature. The slides were washed in water containing 0.04% Photoflow (Kodak) and completely dried at room temperature. The dried spreads were incubated with blocking buffer for 30 min at room temperature. The spreads were then incubated with the indicated primary antibodies listed in Supplementary Table 1 at 4 °C overnight followed by washes with TBS and an incubation with the indicated secondary antibodies for 1 h at room temperature. The slides were observed under a confocal microscope (Nikon).

### Kidney capsule transplantation

FLCs at D11–D13 were collected under a dissecting microscope and cultured in hanging drop with differentiation medium as described above for 2 days. Each hanging drop contained ∼80 FLCs. After 2 days of incubation in the hanging drop, the FLCs formed cell aggregates. These aggregates were then transplanted beneath the kidney capsule of 8–10 week-old bilaterally ovariectomized SCID Beige female mice using a mouth-controlled glass Pasteur pipette as described previously^45^. The recipient mice were dissected 46 days after transplantation, and the grafts were collected for subsequent histological and IF analyses. Mice were maintained under specific-pathogen free conditions, and all the procedures involving animals were performed with the approval of the IRB of the Animal Facility of Tsinghua University.

### *In vitro* maturation of FLCs and drop culture of FLCs

FLCs differentiation from hESC was described above. FLCs on day 11 to day 20 were picked under a dissecting microscope and each FLC was transferred into 20 μl *in vitro* culture medium (IVCM) using 20 μl pipette tips. IVCM was composed of 5% FBS (Gibco), 0.026 M NaHCO_3_ (Sigma), 1 × penicillin and streptomycin solution (Corning), 1 × insulin/transferrin/selenium solution (ITS; Gibco) and 0.01 μg ml^−1^ follicle-stimulating hormone (FSH; R&D) in MEM-α (Corning) as described previously in an *in vitro* maturation study of mice follicles^36^. in brief, each FLC grown on the plate was separated with minimal disturbance to the intact structure, washed twice in IVCM and transferred into 20 μl IVCM attached to the lid of a 60 mm culture dish (Corning). The lid was put in a dish filled with 3 ml of PBS (Corning) to avoid IVCM evaporation. FLC drop culture was incubated at 37 °C in 5% CO_2_. The medium was collected and changed every 24 h.

### Estradiol level detection and measurement

Estradiol concentration was analysed by Estradiol ELA KIT (Cayman Chemical Company, No. 582251). The procedure was performed according to the assay protocol of Estradiol ELA KIT. In brief, eight tubes of standards (4,000 pg ml^−1^, 1,600 pg ml^−1^, 640 pg ml^−1^, 256 pg ml^−1^, 102.4 pg ml^−1^, 41.0 pg ml^−1^, 16.4 pg ml^−1^ and 6.6 pg ml^−1^) were prepared. In one experiment, two wells of blanks, two non-specific binding wells, three maximum binding wells and two wells of standards for each concentration were set on the pre-coated 96-well plate. The plate was incubated at RT for 1 h then washed five times by washing buffer (supplied by the kit). Ellman's Reagent (kit supplied) was added to each well for 1 h at RT in dark. The level of estradiol in each well was measured at 420 nm by ELISA reader (EnSpire Workstation version 4.13.3005.1482) and the concentration of estradiol calculated based on the standard curve.

### Statistical analyses

No statistical method was used to predetermine sample size. All error bars indicate s.d. Prism software was used to perform all statistical analyses of Student's *t*-test, assuming equal variance and a normal distribution, and *P* values <0.05 were considered significant in all analyses. At least three independent experiments were conducted to draw conclusions. The differentiation of hESCs to FLCs had been repeated more than 10 times by multiple researchers using the same protocol to ensure similar outcomes. Each sample size is indicated in the figure legends. No samples or animals were purposely excluded from analyses. No randomization method or blinding was used to allocate samples, animals or researchers during the experiments.

### Data availability

The data that support the findings of this study are available from the corresponding author upon request. The RNA-seq data reported in Fig. 6 have been deposited in the NCBI Gene Expression Omnibus database (http://www.ncbi.nlm.nih.gov/geo/) under the accession code GSE97388.

## Additional information

**How to cite this article:** Jung, D. *et al*. *In vitro* differentiation of human embryonic stem cells into ovarian follicle-like cells. *Nat. Commun.* **8**, 15680 doi: 10.1038/ncomms15680 (2017).

**Publisher's note:** Springer Nature remains neutral with regard to jurisdictional claims in published maps and institutional affiliations.

## Supplementary Material

Supplementary Data 1 Differentially expressed genes of ES, SDE and FLCs

Supplementary Information Supplementary Figures, Supplementary Tables.
