# Supplementary material for: In vitro differentiation of human embryonic stem cells into ovarian follicle-like cells
Source: Nat Commun. 2017 Jun 12;8:15680. doi: 10.1038/ncomms15680 (PMC5472783; doi:10.1038/ncomms15680)
Supplement: Supplementary Information — Supplementary Figures, Supplementary Tables. [file ncomms15680-s2.pdf]

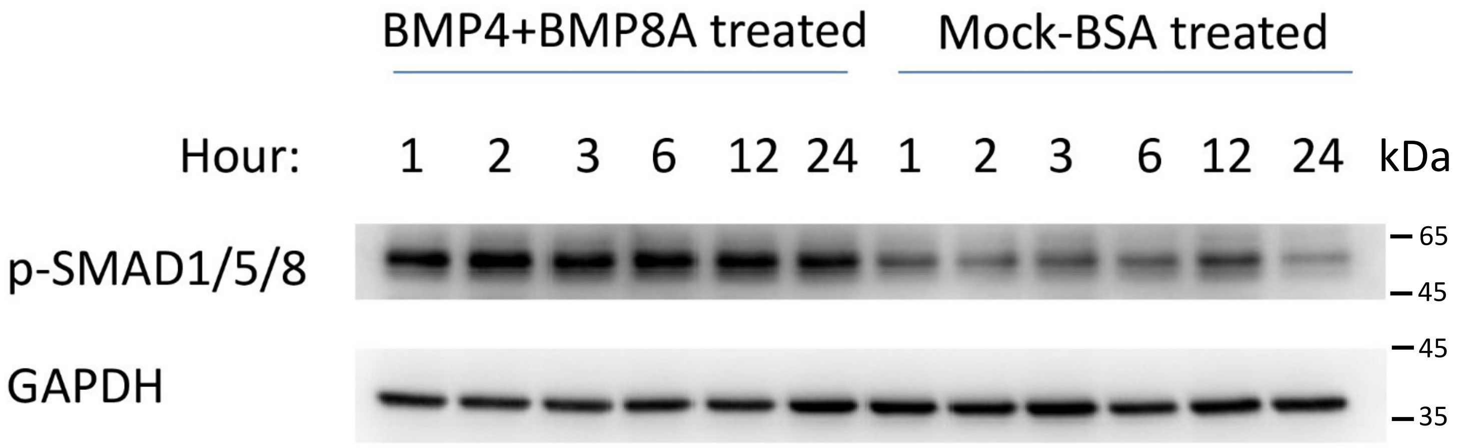

Supplementary Figure 1. Western analysis of p-Smad1/5/8 of differentiated hESCs. H9 hESCs were differentiated with or without BMP4+BMP8A and cell lysates were collected for Western analysis at indicated time point after the treatment. The same amount of protein was loaded for each time point and GAPDH was the loading control.

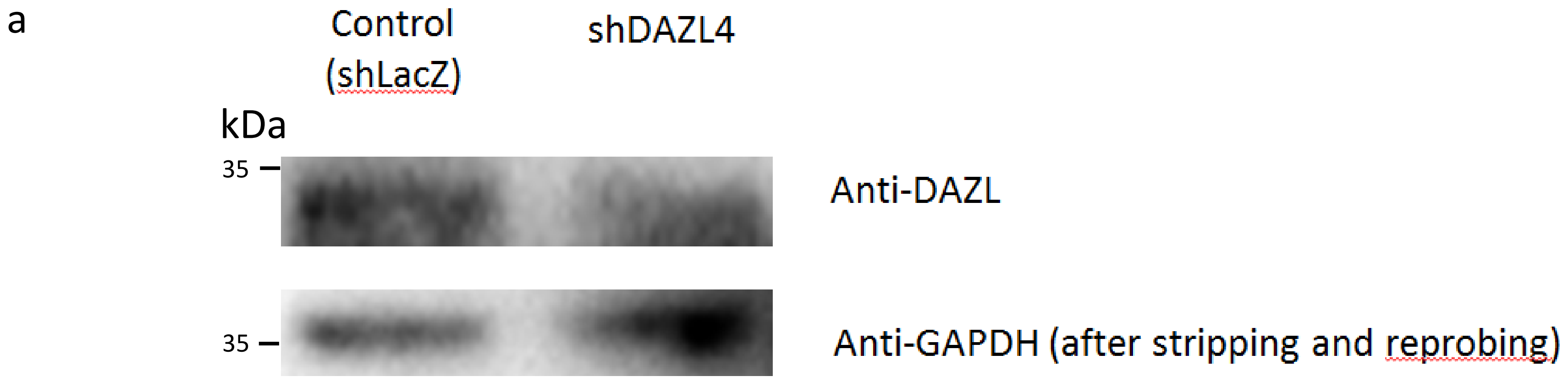

b

ImageJ measurement of intensity

	Area	Mean	Min	Max
DAZL-C	6640	**163.421**	77	238
DAZL-shDAZL	6640	**122.413**	51	198
GAPDH-C	6437	**119.689**	41	196
GAPDH-shDAZL	6437	**188.639**	64	255

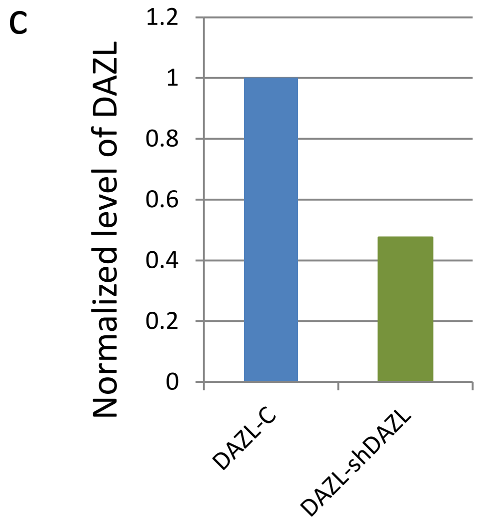

Supplementary Figure 2. Silencing of DAZL in differentiated hESCs . a. Western blots of DAZL and GAPDH. Differentiated hESCs at day6 were subjected to control of silencing vector (shLacZ) and shDAZL4 specifically targeting DAZL transcript. Although the expression of DAZL is low at this differentiated stage, the level of DAZL in the silencing group was reduced to half of the control group, supported by the measurement of intensity using ImageJ (b.) and calculated normalization level of DAZL (c.).

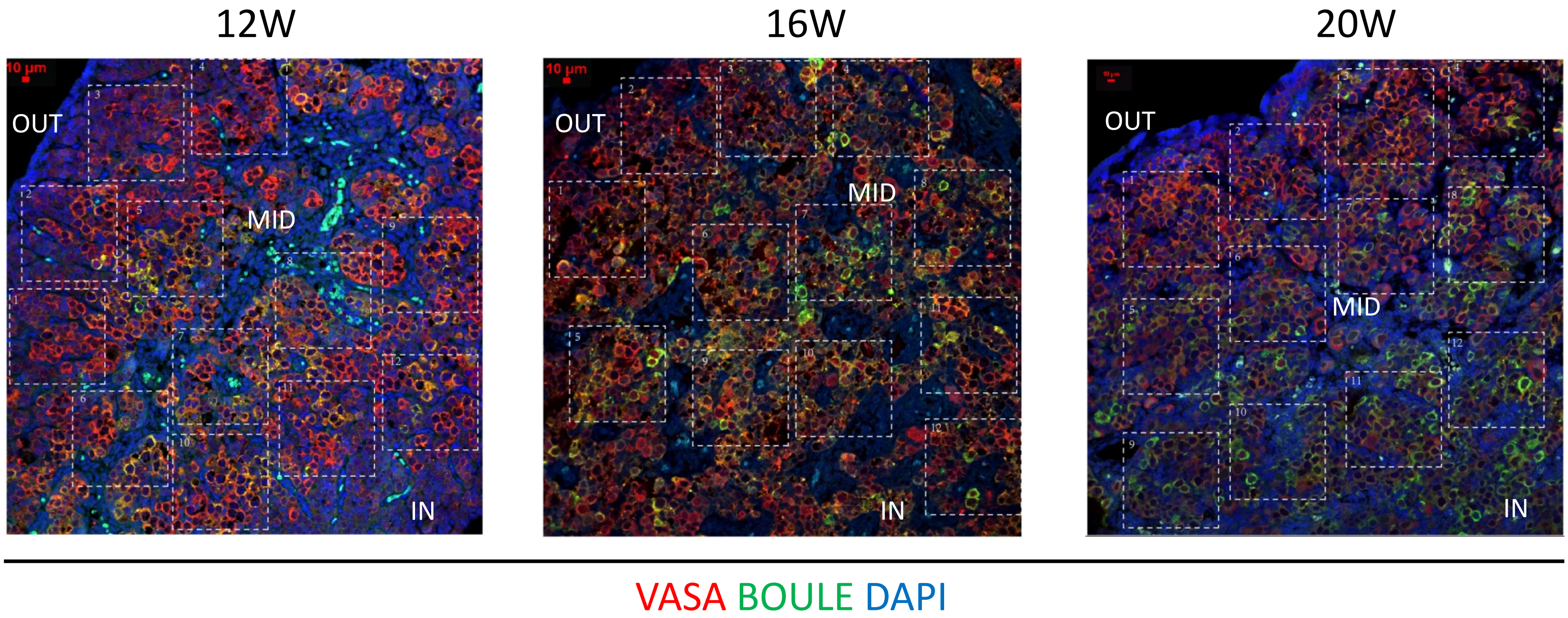

Supplementary Figure 3. Distribution of BOULE positive germ cells in human fetal ovaries. Coimmunostainings of VASA and BOULE were performed on the fetal ovaries of 12W to 20W. The stained sections were divided into 12 different areas, and each area was labelled at the upper left corner. Area 1-4 were resided at the outer cortex, Area 5-8 were resided in the middle cortex and Area 9-12 were resided at the inner cortex. Only VASA positive cells were considered as germ cells and 50-117 germ cells were counted in each area. The percentage of BOULE positive cells were calculated by dividing the total number of BOULE positive cells by the total number of VASA positive cells in each area.

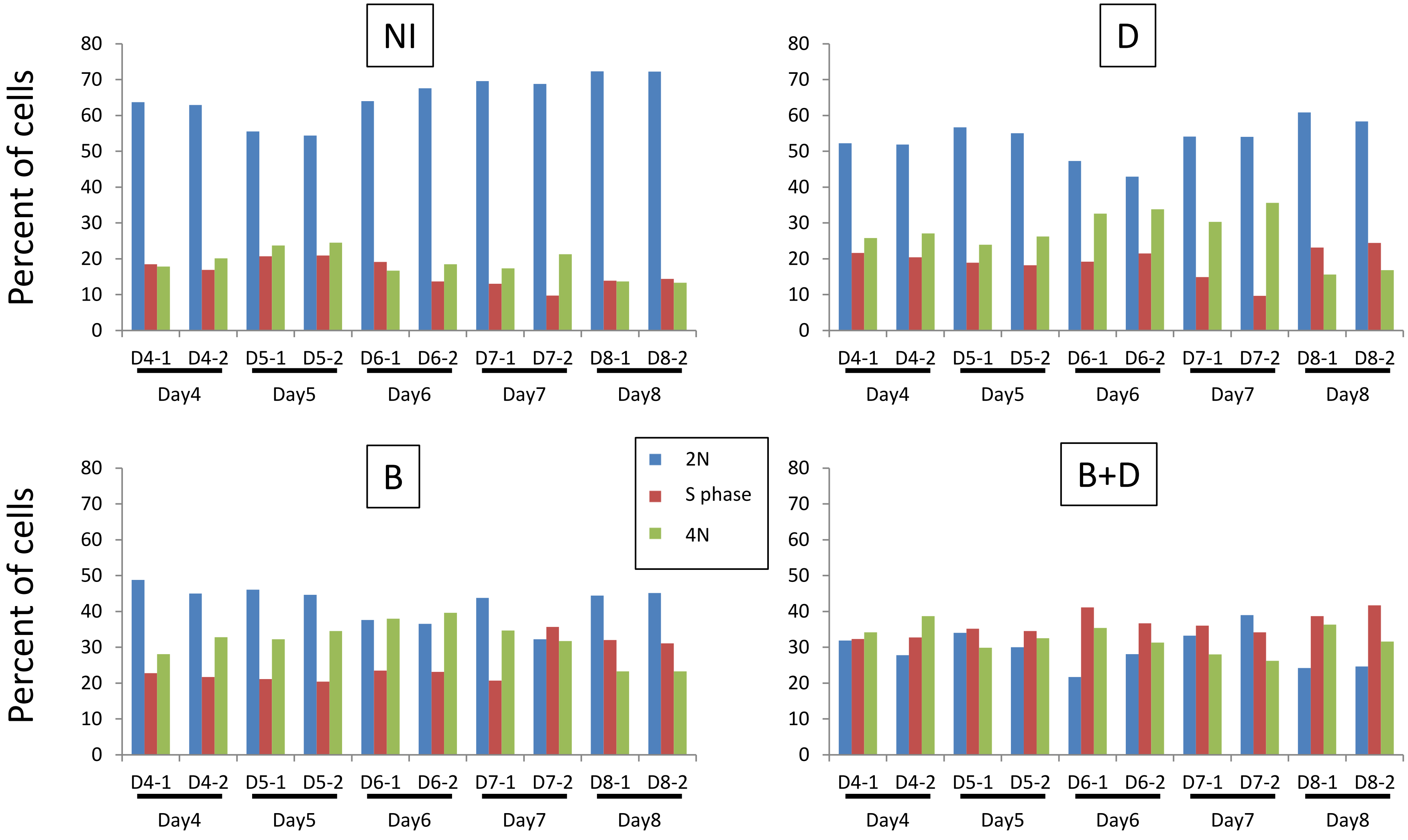

Supplementary Figure 4. Percent of cells with different DNA content. NI, D, B, and B+D cells in 2N, S phase, or 4N from day4(D4) to day8(D8), each sample set had two biological replicates(D4-1 and D4-2) at each time point with >500,000 cells subjected to FACS analysis in each sample.

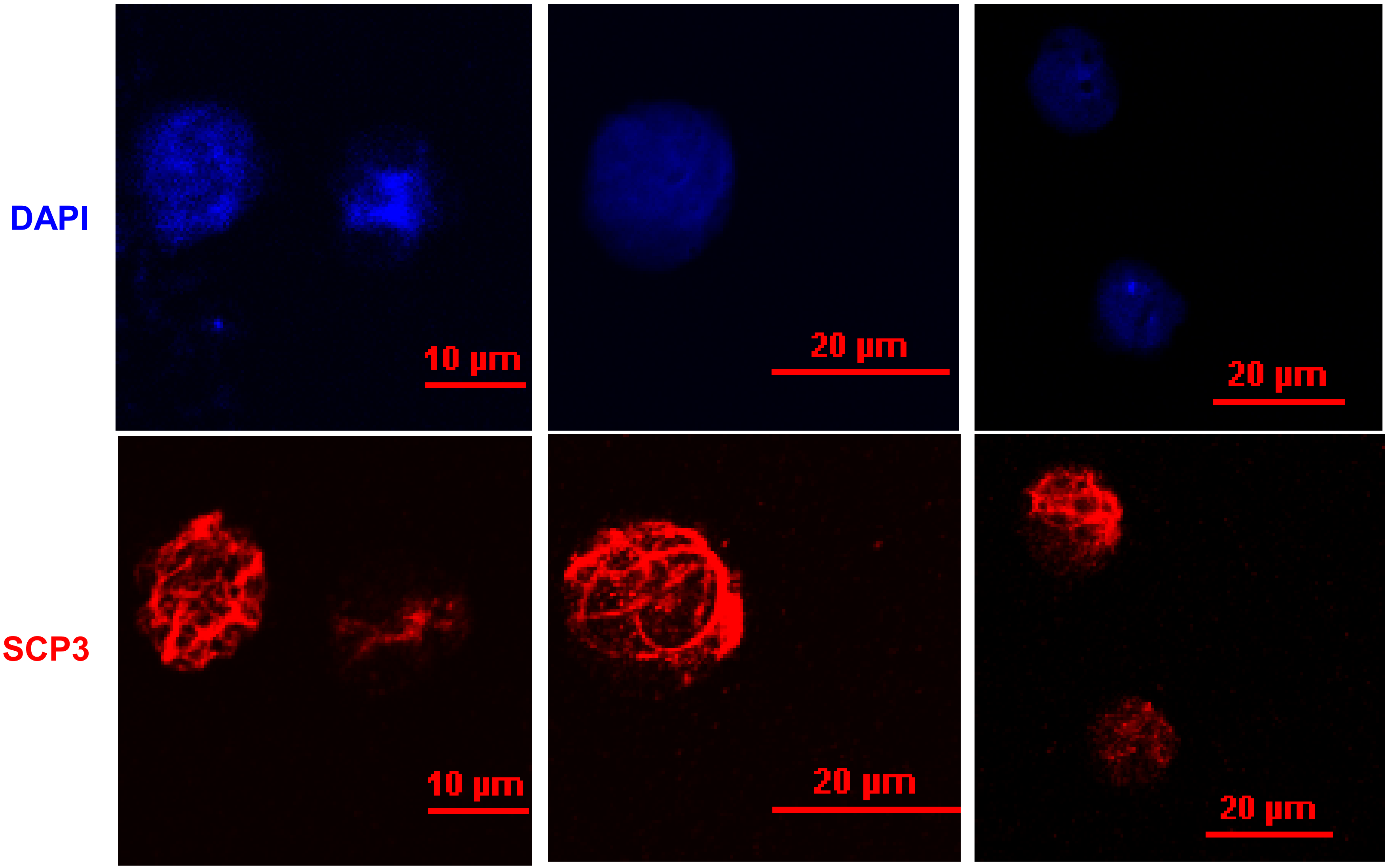

Supplementary Figure 5. SC formation in the induced hESCs. Extensive and elongated SYCP3 stainings can be detected in many induced cells.

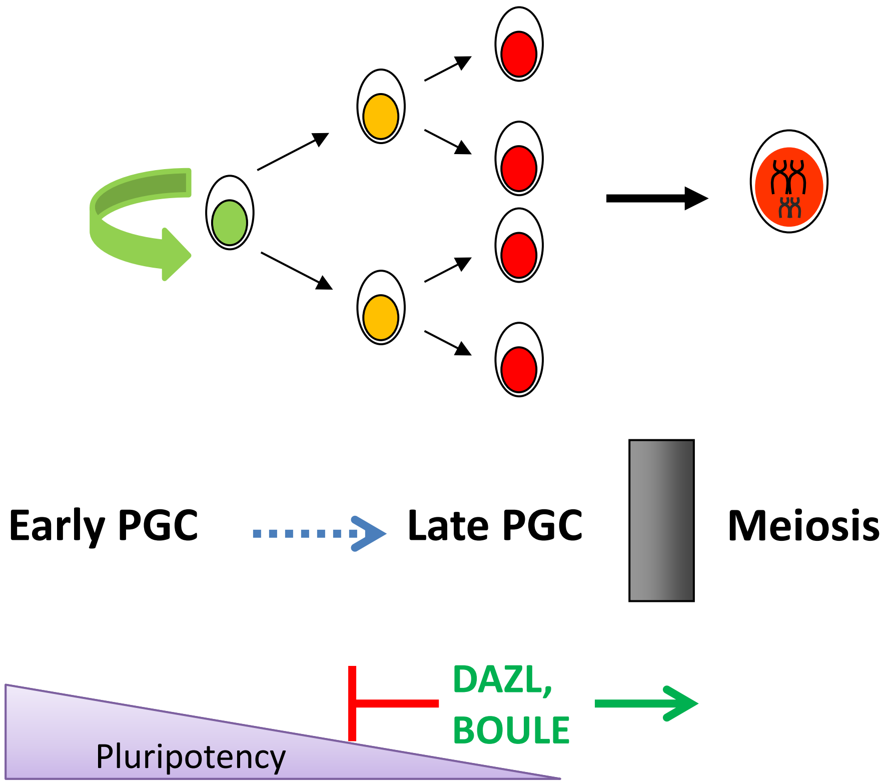

Supplementary Figure 6. Diagram depicting the regulatory roles of DAZL and BOULE in exit of pluripotency and initiation of meiosis during the development of germ cells from pluripotent stem cells.

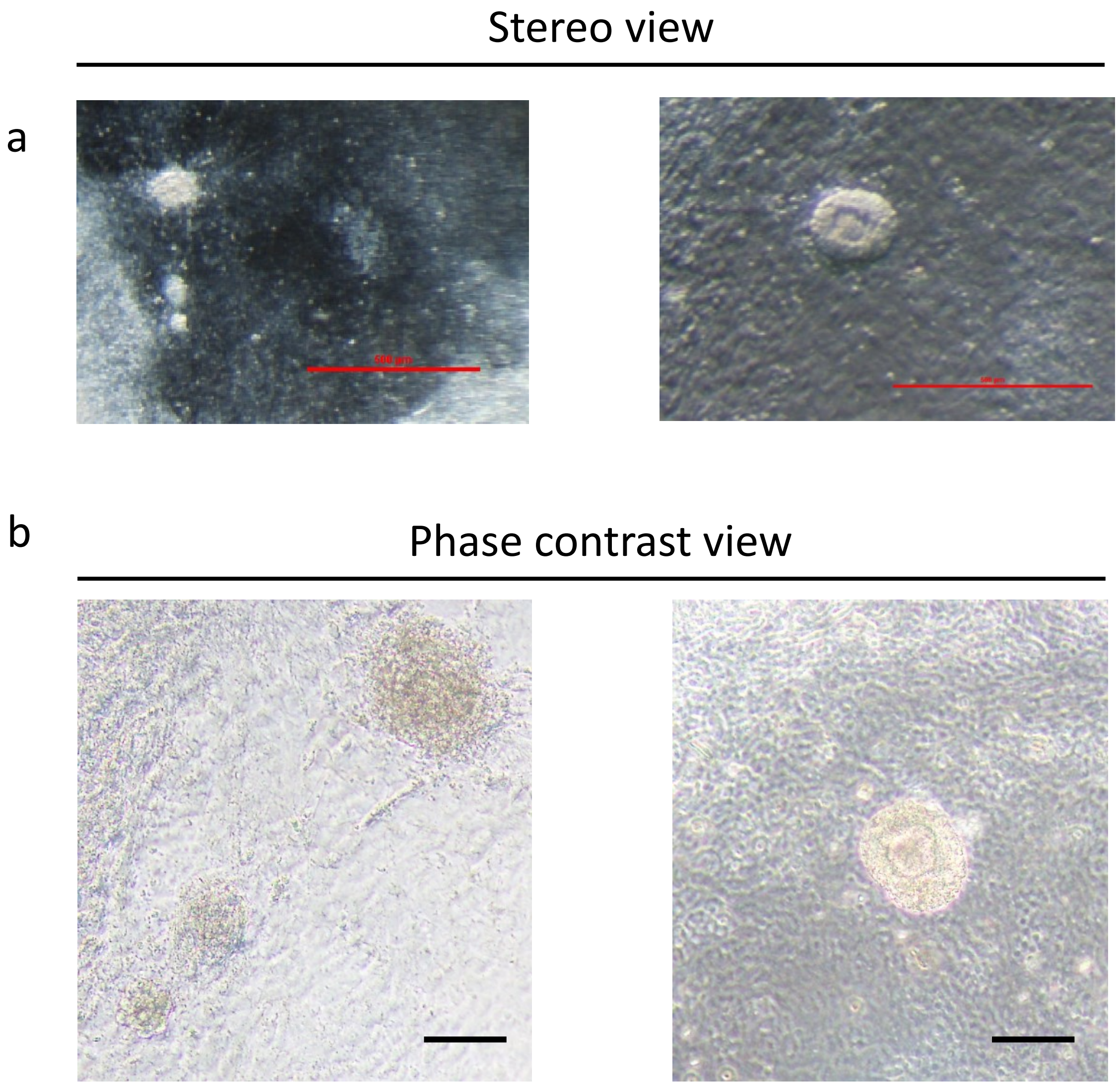

Supplementary Figure 7. FLCs appeared in HSF6 hESC lines. Using the same differentiation protocol as in H9 hESC line, HSF6 line also gave rise to FLCs with morphology resembling ovarian follicles . a. Two independent differentiation experiments recorded by stereo microscope with broader view of the differentiated cells. b. Phase contrast views with higher magnification of the FLCs shown in a.

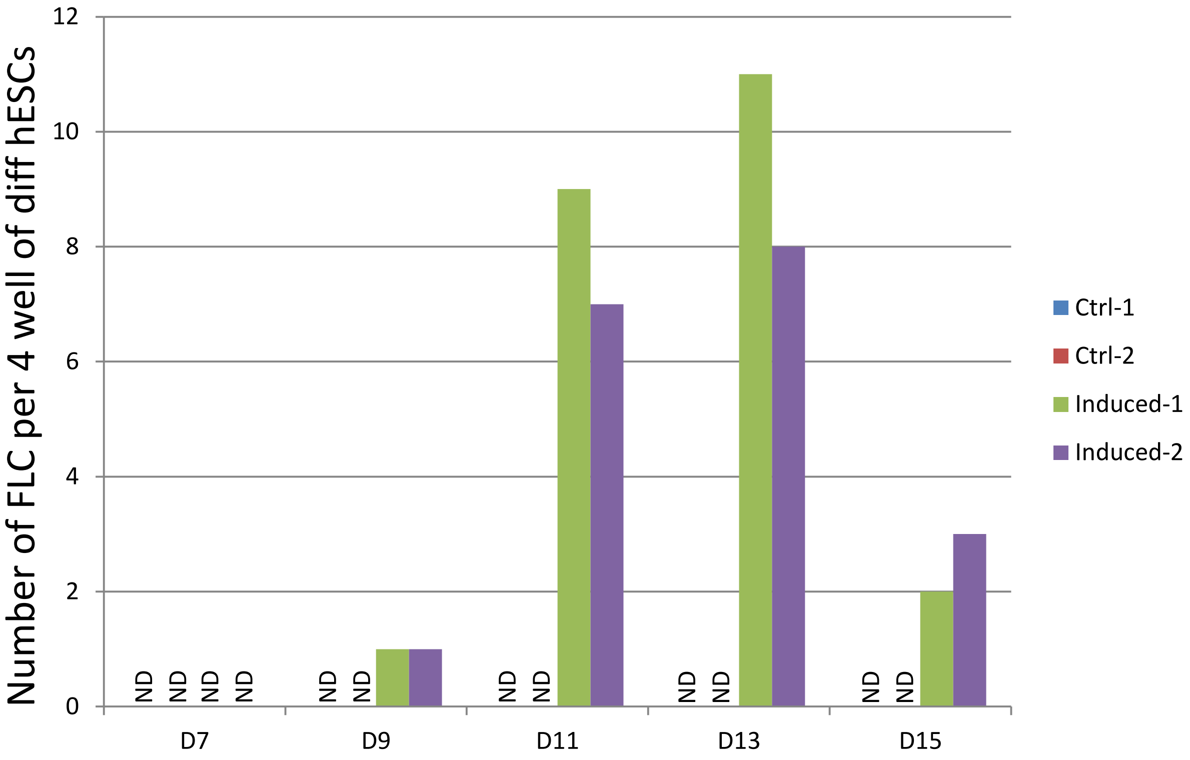

Supplementary Figure 8. Numbers of FLCs observed in the control and induced group from day 7 to day 15, each time point had 2 biological replicates of 4 wells containing approximately 50,000 cells.

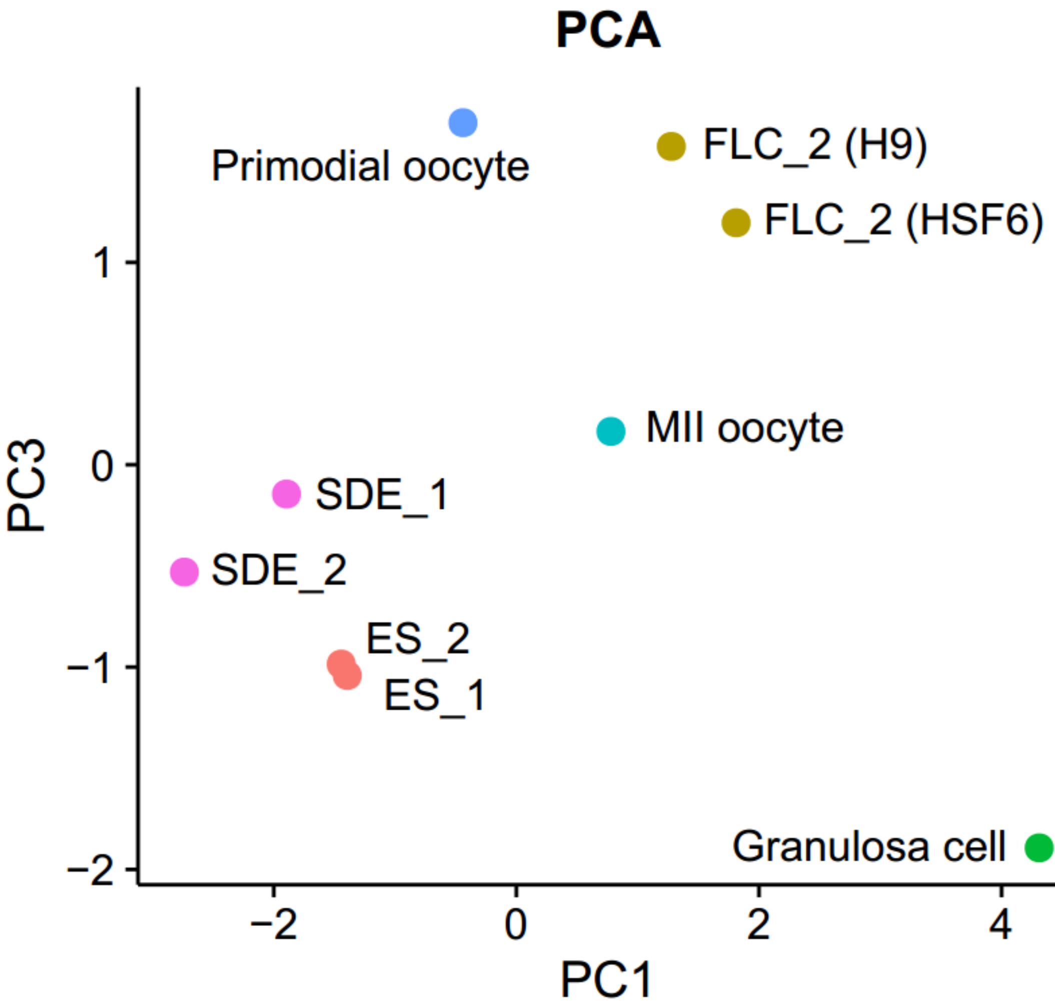

Supplementary Figure 9. Principal component analysis (PCA) of ES, SDE, FLC and primordial oocyte, MII oocyte, granulosa cell from published datasets. We combined the microarray data from in vivo datasets (primordial oocyte, MII occyte and granulosa cells in Grøndahl et al., 2013) and our RNA-seq data (ES, SDE, and FLC) using the overlapping genes, and transformed it to a log2 based value. Then we calculated the z-score across the genes for normalization. PCA analysis were carried out in R using the 'reproduction'-related genes obtained from GO terms. PCA show that FLCs cluster closer to primordial oocytes than MII oocytes and granulosa cells, whereas SDE and ES cluster away from all three in vivo cell types, consistent with our conclusion that FLCs have similar expression pattern of in vivo primordial follicles.

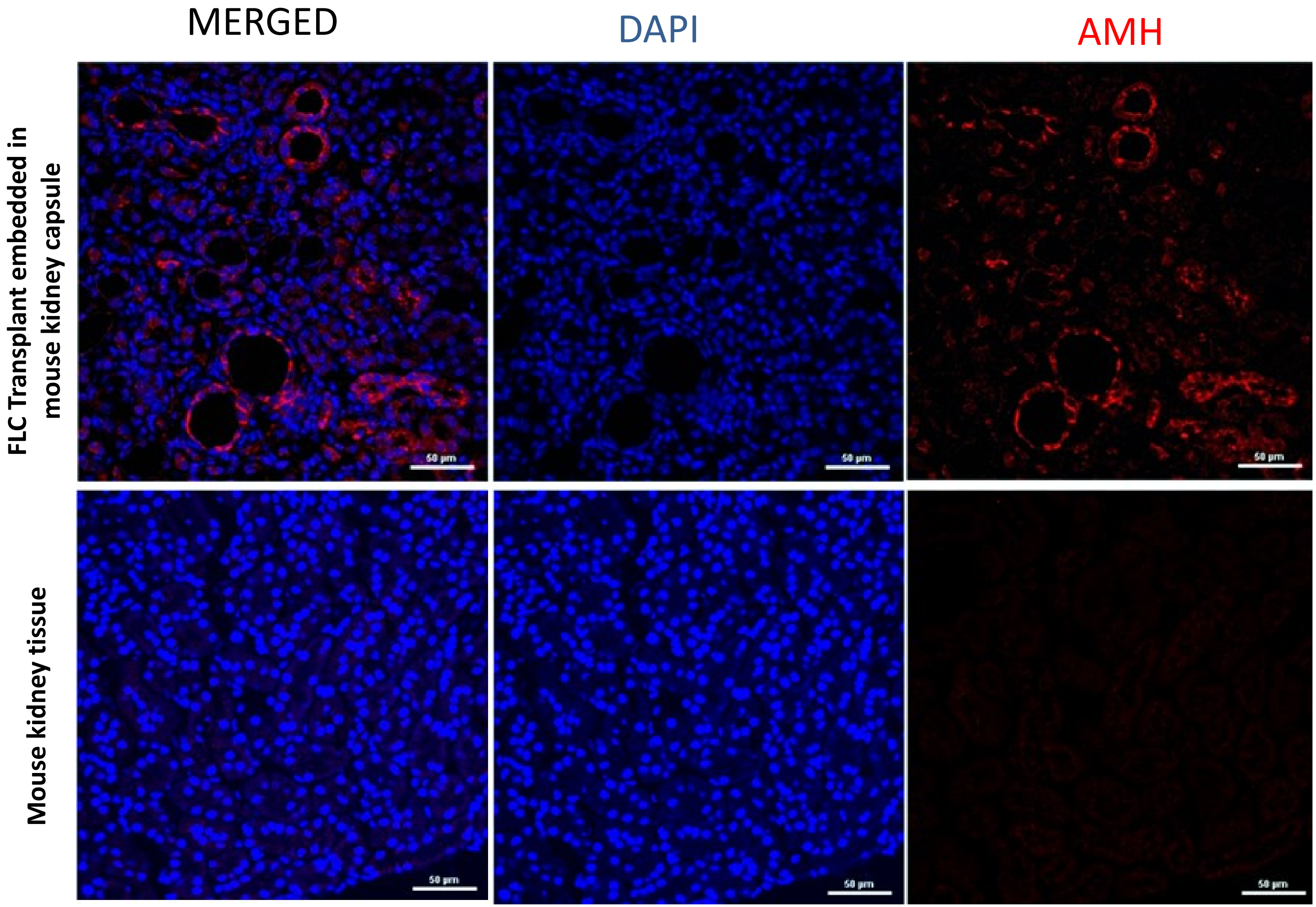

Supplementary Figure 10. AMH positive cells only appeared in transplanted area. Antibody raising against human AMH peptide was used to stain transplanted FLCs and mouse kidney tissue. No specific staining was observed in mouse tissue in contrast with strong signal of human AMH staining in tissue section of transplanted FLCs.

a

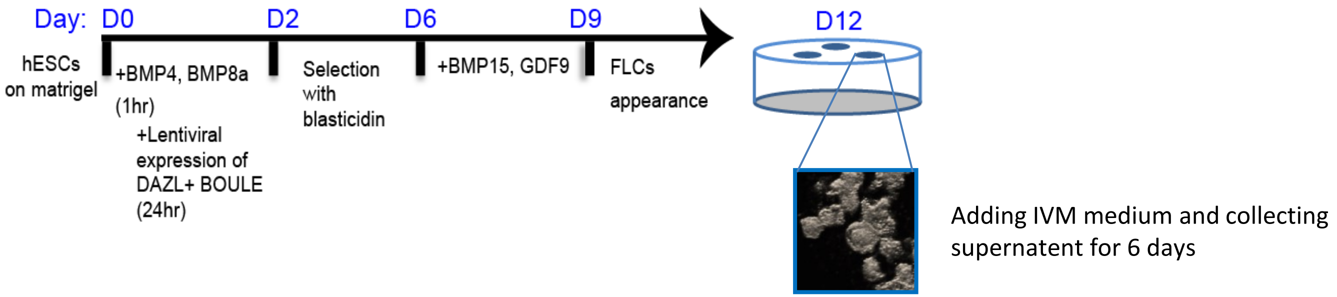

b

Crtl			
**Sample (day)**	**Raw read**	**pg/mL**	**%B/Bo**
H1	0.847	NA	117.9
H2	0.766	NA	105.0
H3	0.785	NA	108.0
H4	0.684	65.4	91.9
H5	0.639	204.3	84.8
H6	0.709	20.8	95.9
H1	0.765	NA	104.8
H2	0.76	NA	104.0
H3	0.755	NA	103.2
H4	0.698	37.7	94.2
H5	0.641	196.4	85.1
H6	0.687	58.9	92.4

FLCs			
**Sample (day)**	**Raw read**	**pg/mL**	**%B/Bo**
H1	1.085	977.4	65.0
H2	1.147	697.9	69.2
H3	1.031	1290.7	61.3
H4	1.06	1113.3	63.3
H5	0.944	1982.9	55.4
H6	1.101	897.8	66.1
H1	1.204	500.2	73.1
H2	1.146	701.8	69.2
H3	1.109	860.1	66.6
H4	1.039	1239.5	61.9
H5	1.091	946.9	65.4
H6	1.027	1945.6	61.1

c

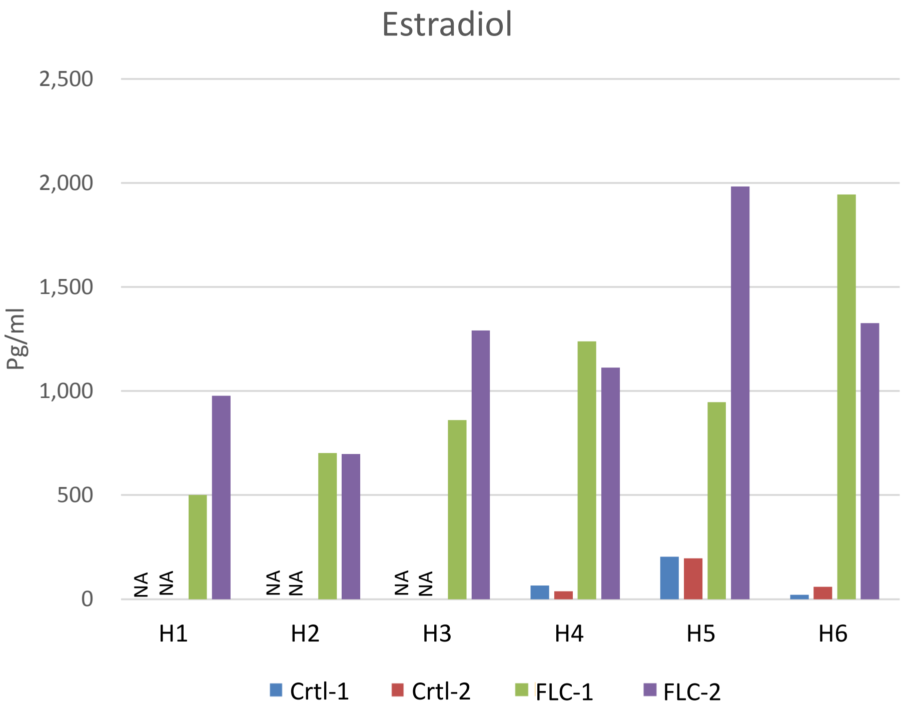

Supplementary Figure 11. Estradiol detection of FLCs in IVM media. a, H9 hESCs were differentiated as depicted in the diagram and FLCs were picked after D11. FLCs were transferred to hanging drop culture containing IVM media and the supernatant was collected each day (H1 to H6). B. Raw readings of estradiol measurements and the concentration of estradiol calculated from standard curve. Control (Ctrl) supernatant collected using spontaneously differentiated hESCs without inducers. Concentration is calculated according to manufacturer's equation : logit(B/Bo)=ln[B/Bo/(1-B/Bo)], taking into account the dilution. The calculated concentration is indicated as NA if the value is out of the detection limit of the assay. %B/BO is an index to indicate if the reading is reliable. %B/Bo above 80 is considered not accurate. c, Graph of the estradiol measurement in b. Each data point was plotted independently as concentration of estradiol. Each time point has two biological replicates of control or FLCs.

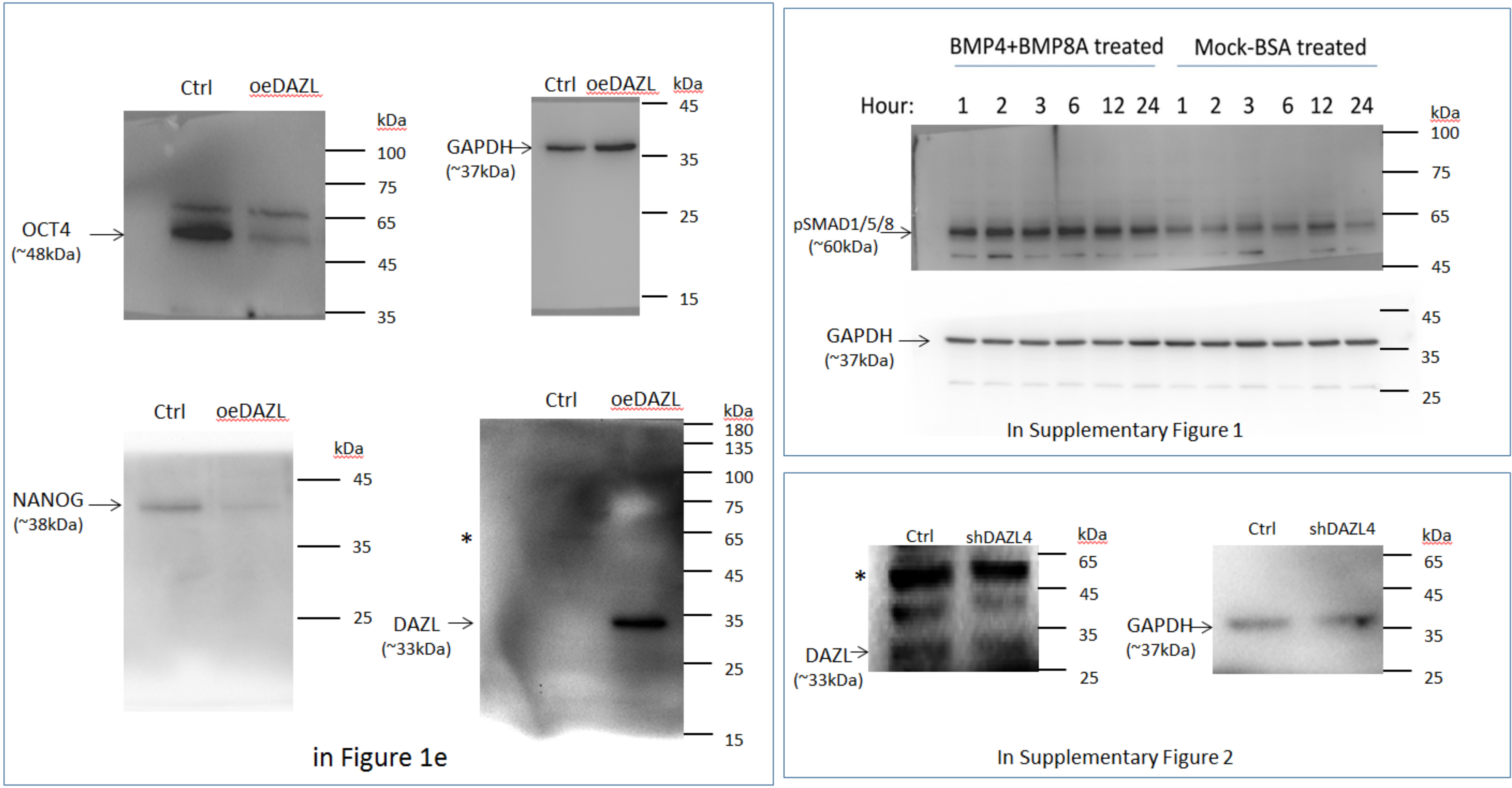

Supplementary Figure 12. Original blots of Western analysis shown in Figure 1e, Supplementary Figure 1 and 2. *: non-specific bands

Supplementary Table 1. Antibodies used in this study

Primary/ Secondary antibodies	Source	Dilution	Cat no.
**DAZL**	Mouse monoclonal	IF, 1:50; Western 1:500	MCA2336, AbD Serotec
**OCT4**	Goat polyclonal	IF, 1:100; Western 1:1000	AF1759, R&D System
**OCT4**	Rabbit polyclonal	1:500	ab19857, Abcam
**BOULE**	Mouse monoclonal	1:100	ab57696, Abcam
**NANOG**	Rabbit polyclonal	1:100	ab21624, Abcam
**p-SMAD1/5/8**	Rabbit polyclonal	Western 1:1000	#9511s, Cell Signaling Technology
**VASA**	Rabbit polyclonal	1:100	ab13840, Abcam
**GAPDH**	Mouse monoclonal	1:5000	60004-1-Ig, Proteintech Group
**ZP2**	Rabbit polyclonal	1:100	sc-30222, Santa Cruz Biotechnology
**NOBOX**	Rabbit polyclonal	1:100	ab41521, Abcam
**AMH**	Rabbit polyclonal	1:100	ab84952, Abcam
**PRDM9**	Rabbit polyclonal	1:100	07-2070-I, Millipore
**γH2AX**	Mouse monoclonal	1:100	ab26350, Abcam
**SYCP3**	Rabbit polyclonal	1:100	NB300-232, Novus
**MLH1**	Mouse monoclonal	1:100	NA28, Calbiochem
**Alexa Fluor 488**	Donkey anti-rabbit	1:1000	A21206, Life technologies
**Alexa Fluor 488**	Donkey anti-mouse	1:1000	A21202, Life technologies
**Alexa Fluor 633**	Donkey anti-goat	1:1000	A21082, Life technologies
**Alexa Fluor 555**	Donkey anti-rabbit	1:1000	A31572, Life technologies

Supplementary Table 2. DNA Primers used in this study.

Gene	Forward Primer	Reversed Primer	Experiment
**GAPDH**	TGTTGCCATCAATGACCCCTT	CTCCACGACGTACTCAGCG	Q-PCR
**OCT4**	AGTGAGAGGCAACCTGGAGA	GTGAAGTGAGGGCTCCCATA	Q-PCR
**NANOG**	TTCCTTCCTCCATGGATCTG	TCTGGAACCAGGTCTTCACC	Q-PCR
**PRDM14**	GAGCCTTCAGGTCACAGAGC	ACCTTCCCACATCTTTCACATC	Q-PCR
**VASA**	AGCTGGGACATTCAATTCGAC	GTTTGGCGCTGTTCCTTTGAT	Q-PCR
**SYCP3**	TATTCCAGGAAATCTGGGAAGCC	GAGCCTTGTTAATGTCAACTCCA	Q-PCR
**CYP19A**	GTGGACGTGTTGACCCTTCT	CAACTCAGTGGCAAAGTCCA	Q-PCR
**SOHLH2**	GGTTGTATTTCAGGGCATGG	CGAACTCTGACAACGAAGCA	Q-PCR
**ZP2**	TCTTCTTCGCCCTTGTGACT	CTCAGGGTGAGCTTTTCTGG	Q-PCR
**NOBOX**	GCCAGAAAGCTGGAGAGAAG	CAGTTCCTCACTCTGAGTGT	Q-PCR
**RSPO1**	AAGTGCTCACCCAAGCTGTT	TTCACATTGCGCAGGACTAC	Q-PCR
**H1FOO**	GTGAAAAAGGCAGCCAAGAG	CTGTAGGCCTCAGCATCTCC	Q-PCR
**CDC25A-3'UTR**	GGGCGGCAGGACCAGCCAGCA	CAGAGCTTCCAACAGTTGGTTAGTA	3'UTR amplification
**CDC25B-3'UTR**	GGGGCCTGCGCCAGTCCTGCTA	CGTGACTCGTTCAACTCTTTGGTCGT	3'UTR amplification
**VASA-3'UTR**	AGCCAAAACATCCTTCAAGTCTG	TAAGGGAAAAGTGTTTCATCCTTTA	3'UTR amplification
**SYCP3-3'UTR**	TGACTCTTTGAAGAAAGAACTTGAACC	TAAAATTAAATCGTCTTTATTTAATTGACAG	3'UTR amplification
